# True-Breeding Targeted Gene Knock-Out in Barley Using Designer TALE-Nuclease in Haploid Cells

**DOI:** 10.1371/journal.pone.0092046

**Published:** 2014-03-18

**Authors:** Maia Gurushidze, Goetz Hensel, Stefan Hiekel, Sindy Schedel, Vladimir Valkov, Jochen Kumlehn

**Affiliations:** 1 Leibniz Institute of Plant Genetics and Crop Plant Research (IPK), Plant Reproductive Biology, Gatersleben, Germany; 2 Institute of Genetics and Biophysics, Naples, Italy; Universidad Miguel Hernández de Elche, Spain

## Abstract

Transcription activator-like effector nucleases (TALENs) are customizable fusion proteins able to cleave virtually any genomic DNA sequence of choice, and thereby to generate site-directed genetic modifications in a wide range of cells and organisms. In the present study, we expressed TALENs in pollen-derived, regenerable cells to establish the generation of instantly true-breeding mutant plants. A *gfp-*specific TALEN pair was expressed via *Agrobacterium*-mediated transformation in embryogenic pollen of transgenic barley harboring a functional copy of *gfp*. Thanks to the haploid nature of the target cells, knock-out mutations were readily detected, and homozygous primary mutant plants obtained following genome duplication. In all, 22% of the TALEN transgenics proved knocked out with respect to *gfp,* and the loss of function could be ascribed to the deletions of between four and 36 nucleotides in length. The altered *gfp* alleles were transmitted normally through meiosis, and the knock-out phenotype was consistently shown by the offspring of two independent mutants. Thus, here we describe the efficient production of TALEN-mediated gene knock-outs in barley that are instantaneously homozygous and non-chimeric in regard to the site-directed mutations induced. This TALEN approach has broad applicability for both elucidating gene function and tailoring the phenotype of barley and other crop species.

## Introduction

The targeted manipulation of genes, involving either their knock-out, replacement or activation, is an attractive technology in the context of both basic and applied genetic research. In a plant breeding context, its major relevance would be to induce mutations specifying novel phenotypes not readily achievable using conventional breeding. A key requirement for such genome engineering is the introduction of double strand breaks (DSBs) in the DNA sequence present at a pre-determined genomic site. Engineered zinc-finger nucleases (ZFNs), transcription activator-like effector nucleases (TALENs), homing endonucleases and RNA-guided nucleases (CRISPRs) have all been reported to be capable of inducing DSBs [1], [2]; of these, the ZFNs have been the most frequently employed. Despite considerable progress made in employing ZFNs for DNA targeting, however, their routine utilization is hampered owing to limited reliability, off-target effects and comparatively high costs. TALENs are customizable fusion proteins between a specific DNA binding domain and FokI endonuclease, the former directing the nuclease to the user-defined genomic site. The binding domain is derived from transcription activator-like (TAL) effectors of pathogenic *Xanthomonas* spp., while the FokI nuclease domain introduces DSBs when present in a dimeric form, achieved by introducing a pair of TALEN units. The resulting DSBs are processed by the host cell's DNA repair machinery, which can leave behind alterations at the breakage sites. As each repeat of the DNA binding domain of the TAL effector recognizes nucleotides in a largely context-independent manner [3], [4], the selection of a target DNA motif and the subsequent customization of the TALEN is made relatively straightforward. Reliable platforms for the modular assembly of TALENs are provided by Golden Gate cloning [5]–[8], high-throughput solid phase assembly [9], and ligation-independent cloning techniques [10].

TALENs have been proven to be highly efficient for targeted genetic manipulation in yeast, human cell lines and animal species. However, their use has been quite limited in plants, including the generation of targeted knock-out mutations in *Nicotiana benthamiana* leaf cells [11], in *Arabidopsis thaliana* [6], [12], in *Brachypodium distachyon* calli and in rice plants [13]. Li et al. [7] have successfully targeted the promoter of a bacterial blight susceptibility gene in rice to generate mutations which converted the host's susceptibility into resistance. A recent demonstration that TALEN-induced DSBs can generate deletions in the promoter of a barley phytase gene has been provided by Wendt et al. [14], but neither the resulting phenotypes nor the sexual transmission of the induced mutations were analysed. Finally, Zhang et al. [15], working with *N. tabacum* protoplasts, achieved TALEN-mediated targeted gene insertion and gene replacement *via* homologous recombination.

A broad spectrum of genomic resources has been developed in barley, along with various enabling technologies such as the large scale production of doubled haploids [16] and effective transformation protocols [17], [18]. The sequence-enriched physical map of barley [19] has provided an invaluable platform for both genome-assisted research and crop improvement. Nevertheless, the targeted modification of specific barley genes remains quite intractable. Establishing usable and efficient protocols to achieve gene targeting would open a number of both basic and applied research areas in this leading temperate cereal species, which is also regarded as a viable model for the genetically more complex, but more important crop species wheat. Here, we describe progress in generating targeted gene knock-outs in barley using TALEN technology. The target chosen to demonstrate the success of the experiments was the reporter gene encoding green fluorescent protein, since this provided a simple bioassay for knock-out events (Fig. S1 in File S1). The TALEN constructs were introduced into embryogenic pollen cultures consisting primarily of haploid cells able to be converted *via* pollen embryogenesis and genome duplication into fertile doubled haploid plants [17]. We show that customized TALENs can induce targeted mutations in barley, and that these are faithfully transmitted to the progeny of primary mutant plants.

## Materials and Methods

### Plant Materials

Grains of non-transformed winter barley (*Hordeum vulgare* L.) cv. ‘Igri’ and of the two transgenic lines PV 89 and BPI 09, which each carry a single copy of *gfp*, were germinated (growth chamber, 14/12°C day/night, 16 h photoperiod 20,000 lux), vernalized for eight weeks (growth chamber, 4°C, 9 h photoperiod) and raised in a glasshouse (18/14°C, 16 h photoperiod at approximately 25,000 lux) to flowering. Spikes were harvested when the awns had just emerged from the sheath of the flag leaf, as described by Kumlehn et al. [17].

### Plasmids and Barley Transformation

A pair of *gfp*-specific TALEN sequences was designed and assembled by Cellectis Bioresearch (Paris, France) using a modified version of *X. campestris* pv. *vesicatoria* AvrBs3 as the backbone for both TALEN units. To evaluate potential off-target cleavage sites in the barley genome (The International Barley Genome Sequencing Consortium, 2012), the kmasker tool (http://webblast.ipk-gatersleben.de/kmasker/) was applied, using standard parameters. The alignments were manually refined, and forty best matching genomic regions were identified.

**Figure 1:**
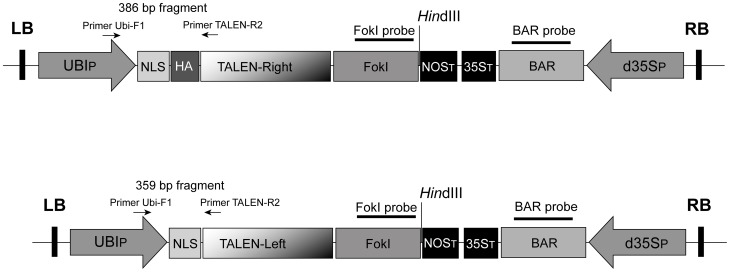
Schematic representation of the T-DNA TALEN constructs. The right-TALEN unit is shown above the left one (not drawn to scale). Primer binding sites are indicated by arrows and the *Fok*I and *BAR* probe regions used for DNA gel blot analysis by bars. LB: T-DNA left border, UBIP: maize *UBIQUITIN-1* promoter, NLS: SV40 nuclear localization signal, HA: hemagglutinin tag, TALEN-Right: customized DNA binding domain of right TALEN unit, TALEN-Left: customized DNA binding domain of left TALEN unit, FokI: DNA cleavage domain of *Fok*I endonuclease, NOST: *A. tumefaciens NOPALINE SYNTHASE* terminator, 35ST: *CaMV 35S* terminator, BAR: bialaphos resistance gene, d35SP: *CaMV 35S* promoter harbouring a doubled enhancer sequence, RB: T-DNA right border, *Hin*dIII: restriction site targeted in the DNA gel blot analysis.

The left (pTAL.pLess.009677) and right (pTAL.pLess.009678) TALEN units were each introduced into the *Spe*I/*Hin*dIII cloning sites of pUbi-AB-M (DNA Cloning Service, Hamburg, Germany) to form an expression cassette under the control of the maize *UBIQUITIN-1* promoter [20]; the *Agrobacterium tumefaciens NOPALINE SYNTHASE* gene provided the termination sequence (*NOST*, Fig. 1). The two intermediate vectors (pUbi-TALEN-Left and pUbi-TALEN-Right) and synthetic sequences encoding the simian virus S40 (SV40) NLS, and NLS-HA (SV40 NLS with an added hemagglutinin tag) were digested with *Asc*I/*Eco*47III, and ligated such that the NLS became attached to the N-terminus of pUbi-TALEN-Left and NLS-HA to the N-terminus of pUbi-TALEN-Right. Each TALEN expression cassette was introduced into the *Sfi*I cloning site of the binary vector p7d35S, which harbours *BAR*, a gene for bialaphos resistance, driven by the cauliflower mosaic virus doubled enhanced *35S* (*CaMV d35S*) promoter [21] (Fig. 1).

Constructs were introduced *via* electroporation into *A. tumefaciens* strain LBA4404pSB1, which carries the disarmed Ti plasmid pAL4404 and the hypervirulence-conferring vector pSB1 [22]. Microspore isolation and the *Agrobacterium*-mediated transformation of embryogenic pollen of cv. ‘Igri’ with the *gfp-*harbouring plasmid pYF133 [23] was carried out as described by Kumlehn et al. [17]. The two *gfp*-specific TALEN sequences were introduced into embryogenic pollen cultures made from lines PV 89 and BPI 09 *via* the same method, except that bialaphos (rather than hygromycin B) was used as the selective agent, since these lines already harboured hygromycin resistance. The density of the transformed LBA4404pSB1 cells was determined spectrophotometrically, and immediately prior to the inoculation the two strains were mixed in a 1∶1 ratio to obtain a final cell density of 2.5×10^7^ per mL.

### Genomic DNA Isolation, PCR, Sequencing and DNA Gel Blot Analysis

A 200–400 mg sample of fresh leaf tissue was snap-frozen in liquid nitrogen, and total genomic DNA was isolated following Palotta et al. [24]. Each 20 μL PCR assay of this DNA was based on 50–100 ng genomic DNA as template, using the sets of primer pairs detailed in Table S1 in File S1. The *gfp* amplicons were purified using a QIAquick PCR purification kit (QIAGEN, Hilden, Germany), and were either directly sequenced, or sequenced following cloning into pGEM-T (PROMEGA, Madison, WI). DNA gel blot analysis was employed to derive the number of integrated T-DNA copies present: for this purpose, a 25 μg aliquot of genomic DNA was digested with either *Hin*dIII or *Ssp*I, electrophoresed through a 0.8% agarose gel and transferred onto a positively charged nylon membrane (Hybond N+, ROCHE, Mannheim, Germany), which was then hybridized with a DIG-labelled probe (*gfp*, *Fok*I or *BAR*) generated from a PCR based on the relevant primer pairs (Table S1 in File S1). Signal was visualized using a DIG System for Filter Hybridisation according to the manufacturer's instructions (ROCHE, Mannheim, Germany).

### RNA Isolation and Reverse Transcriptase Reaction

Total RNA was isolated using the TRIzol reagent (INVITROGEN, Life Technologies, Darmstadt, Germany), following the manufacturer's protocol. After a DNase I treatment (DNA-free kit, AMBION, Life Technologies, Darmstadt, Germany), the cDNA first strand was synthesized using an iScript Select cDNA synthesis kit in the presence of oligo (dT)_20_ primers (BIO-RAD, Munich, Germany). The resulting cDNA served as template for subsequent PCRs based on the *Fok*I domain primer pair (Table S1 in File S1).

### Flow Cytometry and Induced Genome Duplication

The ploidy level of the primary transgenic plants was determined by flow cytometry (Ploidy Analyser 1, PARTEC, Muenster, Germany) according to the manufacturer's instructions. To induce genome duplication (and thereby achieve instant homozygosity of the transgene), the haploid plantlets were treated with 0.1% w/v colchicine, based on a well-established procedure [25].

## Results

### GFP Reporter Lines

To facilitate the detection of site-specific mutagenesis, *gfp* was used as the TALEN target. To this end, single copy transgenic reporter lines were generated *via Agrobacterium-*mediated transformation of cv. ‘Igri’ embryogenic pollen cultures. Instant homozygosity of *gfp* was achieved by treating haploid primary transgenics with colchicine. The number of *gfp* copies present in the progeny of the resulting genome-doubled lines was checked by DNA gel blot hybridization. Two single-copy lines (BPI 09 and PV 89) in which there was no segregation with respect to either the presence (Fig. S2a in File S1) or the expression (Fig. S2b in File S1) of *gfp* were used for the subsequent TALEN experiments.

### Primary TALEN Transgenics and Mutants

**Figure 2:**
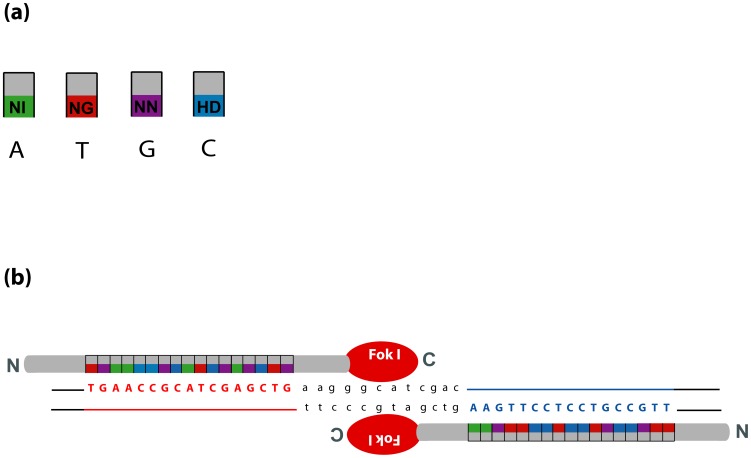
Customized TALENs targeting *gfp*. (a) Four basic modular amino acid repeats with their repeat variable diresidues indicated by the standard single letter codes shown in the colored boxes. The preferred nucleotide recognized by the individual repeat is shown below the repeat. (b) TALENs and their target sequences in the *gfp*. DNA binding specificity was achieved by the arrays of repeats assembled according to the target sequence. The nucleotide sequence shown in red represents the left and the one shown in blue the right-hand TALEN binding site. Lower case letters represent the region where FokI domains are expected to dimerize and introduce DSBs.

**Figure 3:**
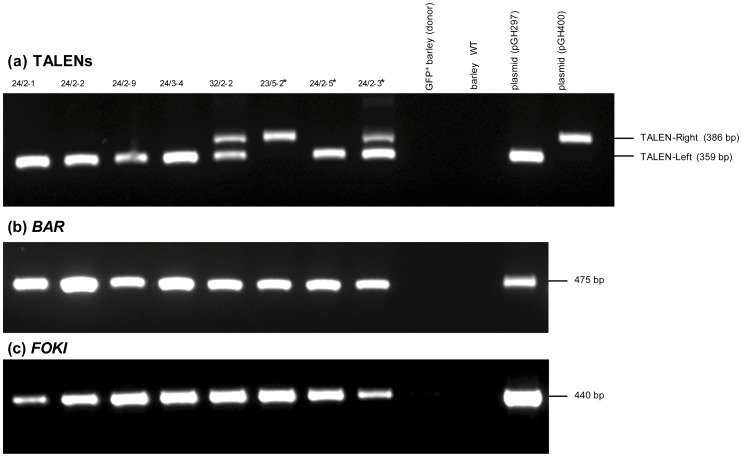
PCR amplification from TALEN transgenics and the parental *gfp* reporter line. Non-mutated transformants marked by asterisks beside their identifier. Amplicons derived from (a) the left (359 bp) and right (386 bp) TALEN units produced using the primer pair Ubi-F1/TALEN-R2 (b) *BAR* (primer pair: GH-Bar-F1/GH-Bar-R1) (c) the *FokI* endonuclease domain (primer pair: FokI-F1/FokI-R). WT: non-transformed cv. ‘Igri’.

**Figure 4:**
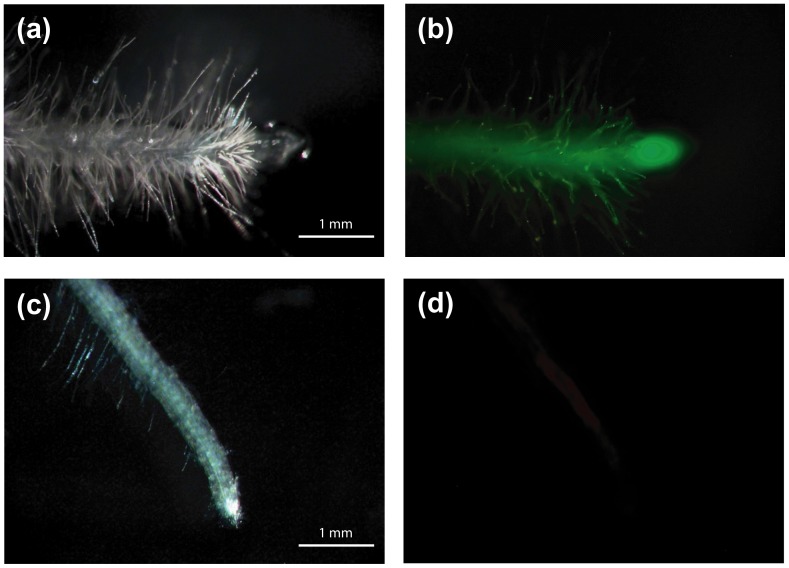
Root tips of reporter and mutated lines visualized. A root tip of a reporter line (a) under white light and (b) when excited with far blue light. A root tip of a TALEN *gfp* knock-out individual (24/2-1) visualized (c) under white light and (d) when excited by far blue light.

**Figure 5:**
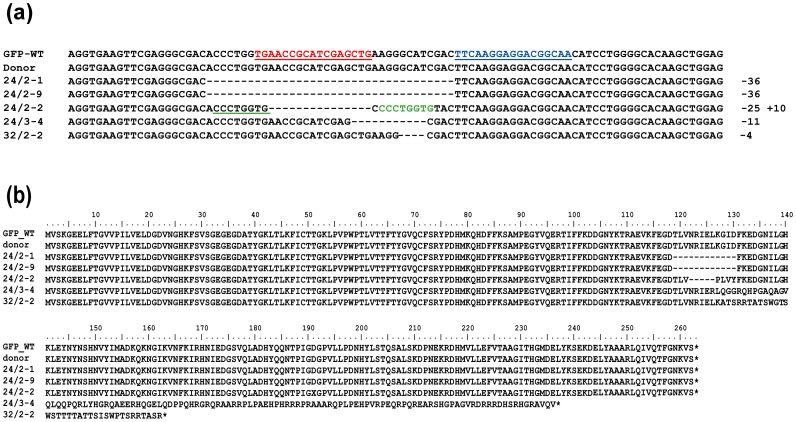
Modifications of the *gfp* sequence induced by TALENs. (a) Alignment of the *gfp* sequences of the various mutants and the donor line. The underlined sequence in red represents the left and that in blue the right TALEN binding site; the sequence shown in green is a duplication of the underlined motif. The number of nucleotides deleted (dashes) or inserted (upper case letters in green) is shown to the right of each sequence. (b) Intact *gfp* (WT) and *gfp* knock-out peptide sequences. Stop codons indicated by asterisks.

**Figure 6:**
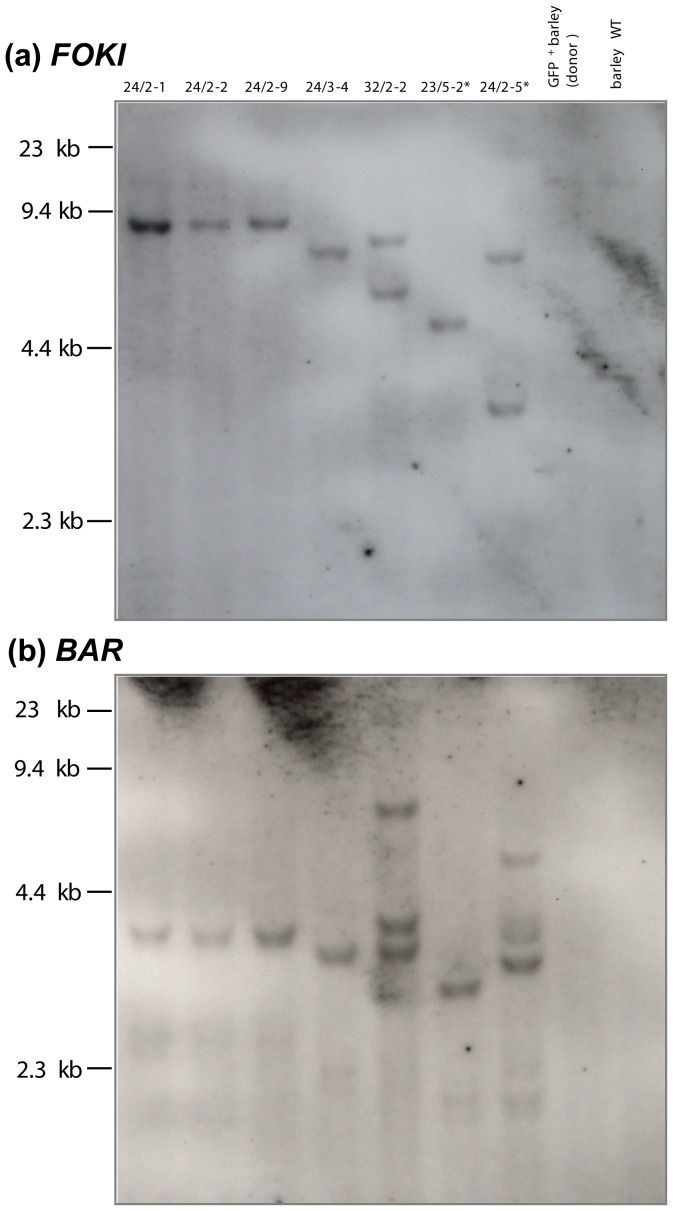
DNA gel blot analysis. Non-mutated transformants marked by asterisks beside their identifier. (a) *Hin*dIII digested DNA hybridized with a *Fok*I probe. (b) *Ssp*I digested DNA hybridized with a *BAR* probe. Note that two copies of *Fok*I and three copies of *BAR* are present in the mutant 32/2-2 suggesting that one of integrated three T-DNA copies is incomplete.

Each TALEN unit comprised 17 modular amino acid repeats specifically targeting selected DNA motifs within *gfp*, along with a C-terminally fused FokI endonuclease domain (Fig. 2). BPI 09 and PV 89 were retransformed with the TALEN constructs, followed by bialaphos selection and the screening of regenerants for the presence of GFP fluorescence, *Fok*I, *BAR* and the TALENs, as well as for *Fok*I expression and ploidy level. Since the *Xanthomonas AvrBs*3 endogenous nuclear localization signals (NLS) had been removed from the standard scaffold to increase TALEN nuclease efficiency [26], the NLS of simian virus S40 was attached to the N-terminus of each TALEN unit. In case of the right TALEN unit, the NLS was combined with a hemagglutinin (HA) tag to facilitate the discrimination between the left and right TALEN units in subsequent molecular analyses (Fig. 1). PCR based on one primer placed within the *UBIQUITIN-1* promoter and the other at the N-terminus of the TALEN sequence yielded a 359 bp amplicon when the left TALEN unit was present, a 386 bp amplicon when the right unit was present and both fragments when both TALEN T-DNAs were successfully incorporated into the host genome (Figs 1, 3a). Of the 18 retransformed regenerants, fourteen proved to carry just the left TALEN unit, two just the right TALEN unit and two harboured both TALEN units (Table S2 in File S1). Four out of the 18 transformants (plants 24/2-1, 24/2-2, 24/2-9 and 24/3-4) did not accumulate GFP (Table 1, Fig. 4). Sequence analysis indicated that each of the *gfp* knock-outs was caused by a genetic modification in the target sequence (Fig. 5), whereas the target sequence in plants still expressing *gfp*, with the exception of 32/2-2, was not modified. The *gfp* amplicon of 32/2-2 was heterogeneous (two different sequences), suggesting that the plant was either heterozygous or chimeric; one sequence was the intact *gfp*, and the other had a 4 nt deletion. PCR analysis showed that 24/2-1, 24/2-2, 24/2-9 and 24/3-4 each possessed stably integrated only the left TALEN T-DNA, while 32/2-2 harboured both the left and right TALEN T-DNA (Fig. 3a), in agreement with the DNA gel blot analysis, which revealed that 24/2-1, 24/2-2, 24/2-9 and 24/3-4 each harboured a single copy of the *Fok*I sequence, while 32/2-2 harboured two copies of the nuclease domain (Fig. 6a). All mutants expressed *Fok*I as was shown using reverse transcription polymerase chain reaction (Fig. S3 in File S1).

**Table 1:** Summary of *gfp*-specific TALEN transformation outputs.

*gfp*-reporter line	Experiment Number	Number of processed spikes	Cultivated pollen [million]	Bialaphos-resistant regenerants	*gfp* mutant alleles	Mutant plant identifier
BPI 09	1	11	1.2	9	3 (2 identical)	24/2-1 24/2-2 24/2-9
BPI 09	2	15	1.6	5	1	24/3-4
PV 89	3	43	4.8	4	1	32/2-2
total	3	69	7.6	18	4 unique	5

The loss of GFP function was caused by deletions ranging in size from 4 to 36 nt. The mutations induced frame shifts and/or the formation of a premature stop codon. Plant 24/2-2 carried a 25 nt deletion plus a 10 nt insertion, where 8 nt of the insertion was result of a duplication of an adjacent sequence (Fig. 5). Apart from plant 32/2-2, at least one of the TALEN binding motifs was altered in the target sequence, thereby abolishing the capability of the TALEN to bind. Overall, five independent targeted mutagenesis events were observed (Table 2). Two events (24/2-1 and 24/2-9) derived from the same transformation experiment, and both carried the same 36 nt deletion.

**Table 2:** Characterization of primary *gfp* knock-out plants.

Mutant identifier	TALEN units stably integrated	*Fok*I copy number^1^	*BAR* copy number^2^	DNA modification (bps)	*Fok*I expression (RT-PCR)	Ploidy of regenerant	Grain set^3^	Zygosity of induced mutation
24/2-1	Left	1	1	−36	+	1n	+	homozygous
24/2-9	Left	1	1	−36	+	1n	+	homozygous
24/2-2	Left	1	1	−25 + 10	+	2n	+	homozygous
24/3-4	Left	1	1	−11	+	1n/2n chimeric	-	not determined
32/2-2	Left + Right	2	3	−4	+	2n	+	heterozygous or chimeric

1 according to DNA gel blot analysis of *Hin*dIII-digested DNA.

2 according to DNA gel blot analysis of *Ssp*I-digested DNA.

3 haploid plantlets treated with colchicine to induce chromosome doubling.

The potential off-targets that might allow forming TALEN hetero- or homodimers were ranked according to the number of mismatches. Forty barley genomic regions that are best matching with the pre-defined TALEN target sequence are shown in Fig. S4 in File S1. The analysis revealed that there is no sequence with less than seven mismatches to the target (Fig. S4 in File S1). Consequently, the presence of an effective off-target in the barley genome is anticipated to be extremely unlikely.

### Heritability of the *gfp* Mutations

**Figure 7:**
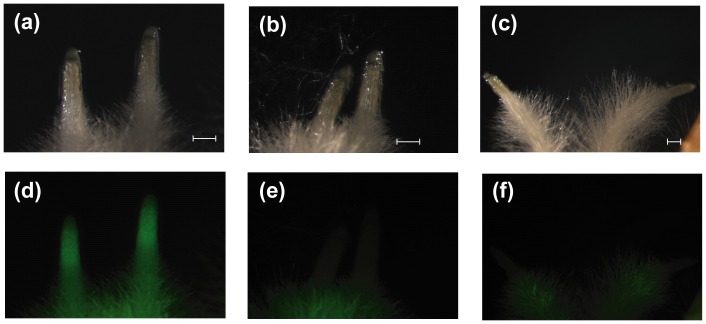
The inheritance of mutated *gfp* in the T_1_ generation bred from plants 24/2-1 and 24/2-2 all of which carried a knock-out copy of *gfp*. Scale bar: 0.2 mm. Root tips imaged (a–c): under bright field, (d–f): after excitation with far blue light. (a, d) *gfp* donor, (b, e) 24/2-1, (c, f) 24/2-2. Some auto-fluorescence was detectable in the root hairs of the mutants (see e, f) and also in those of non-transformed plants (not shown).

**Figure 8:**
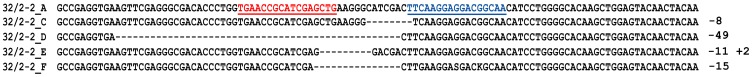
Various *gfp* alleles (A–F) present in T_1_ individuals derived from the primary transformant 32/2-2. The underlined sequence in red represents the left and that in blue the right TALEN binding site. Note that allele 32/2-2_B (not shown in this figure) that was identified in the primary mutant could not be detected in the progeny.

Except for plant 24/3-4, each of the TALEN transgenics grew normally and were self-fertile following either colchicine treatment-induced or spontaneous chromosome doubling. The mutant 24/3-4 was a ploidy chimera involving both 1n and 2n sectors. Apparently only haploid rather than the diploid tissue contributed to the germ line causing sterility of this mutant. This phenomenon is not unusual for regenerants derived from embryogenic pollen cultures [27], [28]. Respectively, the transmission of the mutation was determined for the colchicine-treated doubled haploid 24/2-1 and the spontaneously diploidized mutants 24/2-2 and 32/2-2. The zygosity of the primary transgenic plants were determined by scoring for GFP fluorescence in the pollen (Fig. S5 in File S1) and in T_1_ seedlings (Fig. 7). In addition, the *gfp* sequence was acquired from a sample of T_1_ individuals to identify whether the T_0_ mutants carried more than one independent sequence alteration. At least two grains per spike were germinated, resulting in sets of 21, 46 and 32 T_1_ individuals derived from 24/2-1, 24/2-2 and 32/2-2, respectively. In the cases of 24/2-1 and 24/2-2, every T_1_ individual carried the same mutated *gfp* sequence as did their primary transformant parent, and none expressed functional *gfp.* Whereas in the progeny of 32/2-2, segregation with regard of GFP fluorescence was detected. Furthermore, sequencing of the *gfp* in the progeny of the primary mutant 32/2-2 showed that various new *gfp* alleles were present in the T_1_ that had not been detected in the parental plant. In addition, several T_1_ plants carried the intact (wild-type) *gfp* allele. The new mutant *gfp* alleles observed in the T_1_ possessed indels at the TALEN target site (Fig. 8). Notably, the mutant allele recovered in the T_0_ primary mutant 32/2-2 (4 nt deletion) was no longer identified among the sequenced T_1_ individuals.

## Discussion

### TALEN-Mediated Gene Modification in Barley

Thanks to the combination of targeting *gfp* and exploiting haploid technology, it was possible to provide unequivocal evidence for targeted gene knock-out in barley. We have demonstrated for the first time that customizable designer nucleases can be expressed and function in haploid cells, which in turn can be instantly regenerated into homozygous mutants. The sample of mutants showed that the altered alleles were faithfully transmitted through meiosis and so were passed on to the sexual progeny of the primary transgenics.

The recent acquisition of the barley genome sequence (The International Barley Genome Sequencing Consortium, 2012) largely facilitates the identification of appropriate target sites within genes known to determine traits of interest to barley breeders, and offers the possibility of excluding (or at least minimizing) off-target effects and achieving a high specificity of the designed nucleases. We screened the barley genome for potential off-target cleavage sites. However, even the best matches we found contained at least seven mismatches compared to the TALEN target nucleotide sequence (Fig. S4 in File S1). Recent studies showed an extremely low off-target activity even if only one nucleotide mismatch was present [15], [29]. Considering this high specificity of TALENs, we did not find any barley genomic sequences that could be seriously considered as potential off-targets. In conclusion, we assume that the unintended induction of mutations in the barley genome by the *gfp*-specific TALENs is at least indistinguishable from the occurrence of background mutations.

The frequency with which mutation was induced (∼20%) and the characteristics of the induced mutations were comparable to what has been documented in other examples of the TALEN approach [12]–[14]. Two cases of rather larger deletions were observed, one involving the loss of 36 nt, including the entire left TALEN binding site, and the other of 25 nt which was accompanied by a 10 nt insertion. This sort of modifications has been associated with the repair of DSBs *via* non-homologous end joining. Most modifications induced were deletions, which is in accord with the observations made by Kim et al. [30] who reported that in contrast to the modifications induced by ZFNs, most TALEN events are deletions, and speculated that the reason for this may be associated with the comparatively large spacer region provided between the two TALEN binding sites.

Four of the five primary mutant plants showed no evidence of heterozygosity for the *gfp* sequence, while the remaining one (32/2-2) harboured two distinct *gfp* sequences. The presence of two different *gfp* alleles in the latter primary transgenic implies that the plant was either heterozygous or chimeric. TALEN-induced mosaicism involving as many as nine variant sequences of the *HvPAPhy_a* promoter region in a single barley primary transgenic plant has recently been recorded [14]. No such extreme cases of multiple sequences were observed here, suggesting that the use of embryogenic pollen as the explant encourages the formation of single, rather than multiple targeted modification events and that the TALENs preferentially induce mutations at the early stage of embryogenic development. Our hypothesis is that the early disruption of the TALEN binding sites avoids repeated binding of the TALENs, thereby ensuring that no further diversification of mutations will be induced in subsequent daughter cells.

The two primary mutants 24/2-1 and 24/2-9 carried an identical 36 nt deletion. The repeated recovery of specific designer endonuclease-derived modifications has been reported in maize [31], zebrafish [32], and rat [33]. The latter authors have suggested the possibility that microhomology-mediated reclosure of DSBs could explain the phenomenon. In the present case, however, the same deletion was recovered from the same experiment, which would suggest that the two individuals were in fact clones derived from a single mutagenesis event.

More notable was the finding that four of the five induced mutants possessed only a single stably integrated TALEN unit, as confirmed by DNA gel blot analysis. The creation of DSBs requires FokI dimerization [34], [35], so the assumption is that the non-integrated TALEN unit must have only been transiently expressed in the cells of embryogenic pollen from which these mutants were derived. If so, the implication is that the transient expression of TALENs can induce DSBs at a very early stage of pollen embryogenesis. A similar non-integration of a TALEN construct has recently been observed in experiments conducted using tobacco protoplast-derived calli [15]. The possibility that whole plants harbouring targeted mutations can be generated from cells modified by the transient expression of TALENs offers the prospect of inducing targeted mutations and transgene-free mutants *via* non-integrative expression. In other words, it may be feasible to avoid the laborious procedure of removing the TALEN-coding expression cassettes by segregation. In this context, it is significant that TALENs can act as a mutagen *in planta* without necessarily requiring the maintenance of recombinant DNA. In this sense, the use and regulation of mutant crop plants generated *via* TALENs may not be as troublesome as those which are legislatively classed as being genetically engineered.

### The Transmission and Zygosity of the Mutations

The transmission of the *gfp* mutations through meiosis was explored in T_1_ progenies derived from 24/2-1, 24/2-2 and 32/2-2. The reason for the choice of the primary mutant 24/2-1 was that it was a haploid mutant regenerant treated with colchicine to induce chromosome doubling, which ensured that the plant was homozygous for the *gfp* mutation. In contrast, 24/2-2 and 32/2-2 were spontaneously chromosome doubled plants, so could have been either homozygous or heterozygous with regard to the *gfp* allele disruption, depending on whether the mutation event occurred prior to or after diploidization. The progenies 24/2-1 and 24/2-2 were not showing any segregation for GFP accumulation, either among their pollen or among their selfed offspring. The conclusion is that the *gfp* mutation must have occurred in a haploid cell in both cases. The ability to mutagenize haploid cells permits the generation of instantly homozygous mutants, which is highly advantageous compared to previously published methods of gene targeting. It has also been possible to show that the altered *gfp* alleles were transmitted through meiosis to the gametophyte and hence to the progeny of primary mutants, just as has been demonstrated in rice in the case of an altered *Os11N3* promoter sequence [7]. Among the progeny of 32/2-2, multiple *gfp* alleles were observed that had not been detected in the T_0_ primary mutant. On the other hand, the allele present in the primary mutant was not recovered among 32 sequenced T_1_ individuals. The reason might be that the T_0_ primary mutant was chimeric concerning the mutant alleles present, and the cells harbouring the observed allele (4 nt deletion) did not contribute to the germline of the primary mutant. Alternatively, as the primary transgenic mutant 32/2-2 carried both TALEN units stably integrated, the TALENs might have kept binding the target sites repeatedly producing new mutant alleles until at least one of the target sites had been destroyed, that rendered the target region inaccessible to the TALE nucleases.

In conclusion, we have demonstrated a practical method for inducing heritable targeted gene disruption in barley using TALENs. This success should encourage the application of this technology to elucidate gene function and develop improved or new traits not only in barley, but also in other cereal species, in particular wheat and maize. The results obtained may also pave the way for the establishment of ways to use TALENs to edit cereal and other crop plant sequences based on DSB repair *via* homologous recombination.

## Supporting Information

File S1 **Figures S1–S5 and Tables S1–S2.** Figure S1: Embryogenic pollen cultures developed from donor *gfp* transgenic barley. Figure S2: DNA gel blot and *gfp* expression analysis of the donor line PV89. Pollen culture of independent hemizygous transgenic plant is shown as a control. Figure S3: PCR amplification of the *Fok*I domain from a template of cDNA prepared from each of the primary *gfp* mutants, the *gfp* donor and non-transformed cv. ‘Igri’. *ACTIN* served as a positive control. Figure S4: Best matches found in the barley genome as compared to the target sequence. Figure S5: Immature pollen produced by primary transgenic *gfp* knock-out plant 24/2-1 and *gfp* donor. Table S1: Primer sequences used for the identification of T-DNA elements. Table S2: Transgenic plants characterized for ploidy level and the presence and expression of TALEN T-DNA. Sequences of the used binary vectors.
(DOC)
