# Supplementary material for: True-Breeding Targeted Gene Knock-Out in Barley Using Designer TALE-Nuclease in Haploid Cells
Source: PLoS One. 2014 Mar 18;9(3):e92046. doi: 10.1371/journal.pone.0092046 (PMC3958423; doi:10.1371/journal.pone.0092046)
Supplement: File S1 — Figures S1–S5 and Tables S1–S2. Figure S1: Embryogenic pollen cultures developed from donor gfp transgenic barley. Figure S2: DNA gel blot and gfp expression analysis of the donor line PV89. Pollen culture of independent hemizygous transgenic plant is shown as a control. Figure S3: PCR amplification of the FokI domain from a template of cDNA prepared from each of the primary gfp mutants, the gfp donor and non-transformed cv. ‘Igri’. ACTIN served as a positive control. Figure S4: Best matches found in the barley genome as compared to the target sequence. Figure S5: Immature pollen produced by primary transgenic gfp knock-out plant 24/2-1 and gfp donor. Table S1: Primer sequences used for the identification of T-DNA elements. Table S2: Transgenic plants characterized for ploidy level and the presence and expression of TALEN T-DNA. Sequences of the used binary vectors. (DOC) [file pone.0092046.s001.doc]

**SUPPORTING INFORMATION**

**True-breeding targeted gene knock-out in barley using designer TALE-nuclease in haploid cells**

*Maia Gurushidze^1,2^, Goetz Hensel^1^, Stefan Hiekel^1^, Sindy Schedel^1^, Vladimir Valkov^1,3^ and Jochen Kumlehn^1^*

*^1^* Leibniz Institute of Plant Genetics and Crop Plant Research (IPK), Plant Reproductive Biology, Corrensstrasse 3, 06466 Gatersleben, Germany

^3^Institute of Genetics and Biophysics, Via Pietro Castellino 111, 80131 Naples, Italy

*^2^* Corresponding author:

Maia Gurushidze

E-mail: [maiko@ipk-gatersleben.de](mailto:maiko@ipk-gatersleben.de)

Phone: +49 (0) 39482 5357

Fax: +49 (0)39482 5515

**Keywords**

*Hordeum vulgare*, gene targeting, homozygous mutants, GFP, pollen cultures, haploid technology


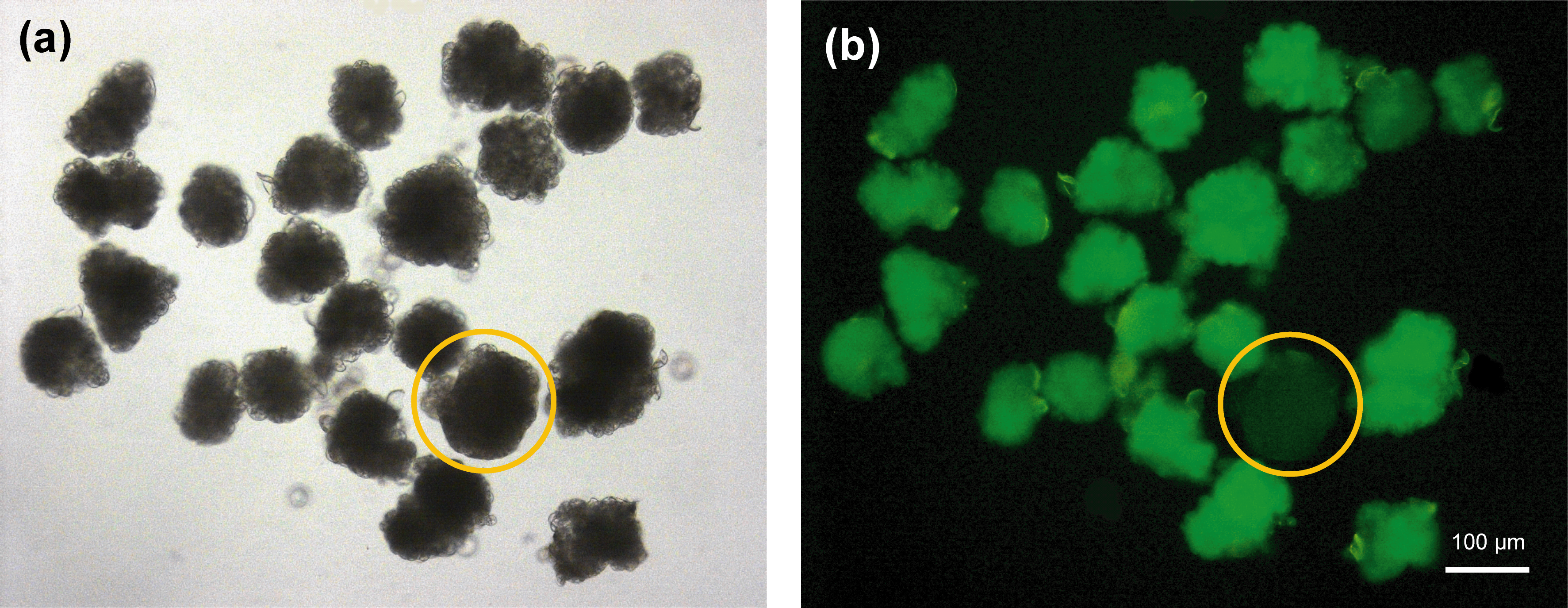


Figure S1. Embryogenic pollen cultures developed from donor *gfp* transgenic barley. Immature pollen-derived calli visualized (a) under bright field light, (b) following excitation by far blue light. The presence of a non-fluorescent callus (circled in yellow) is readily identified; such calli develop from pollen cells in which *gfp* has been knocked out.


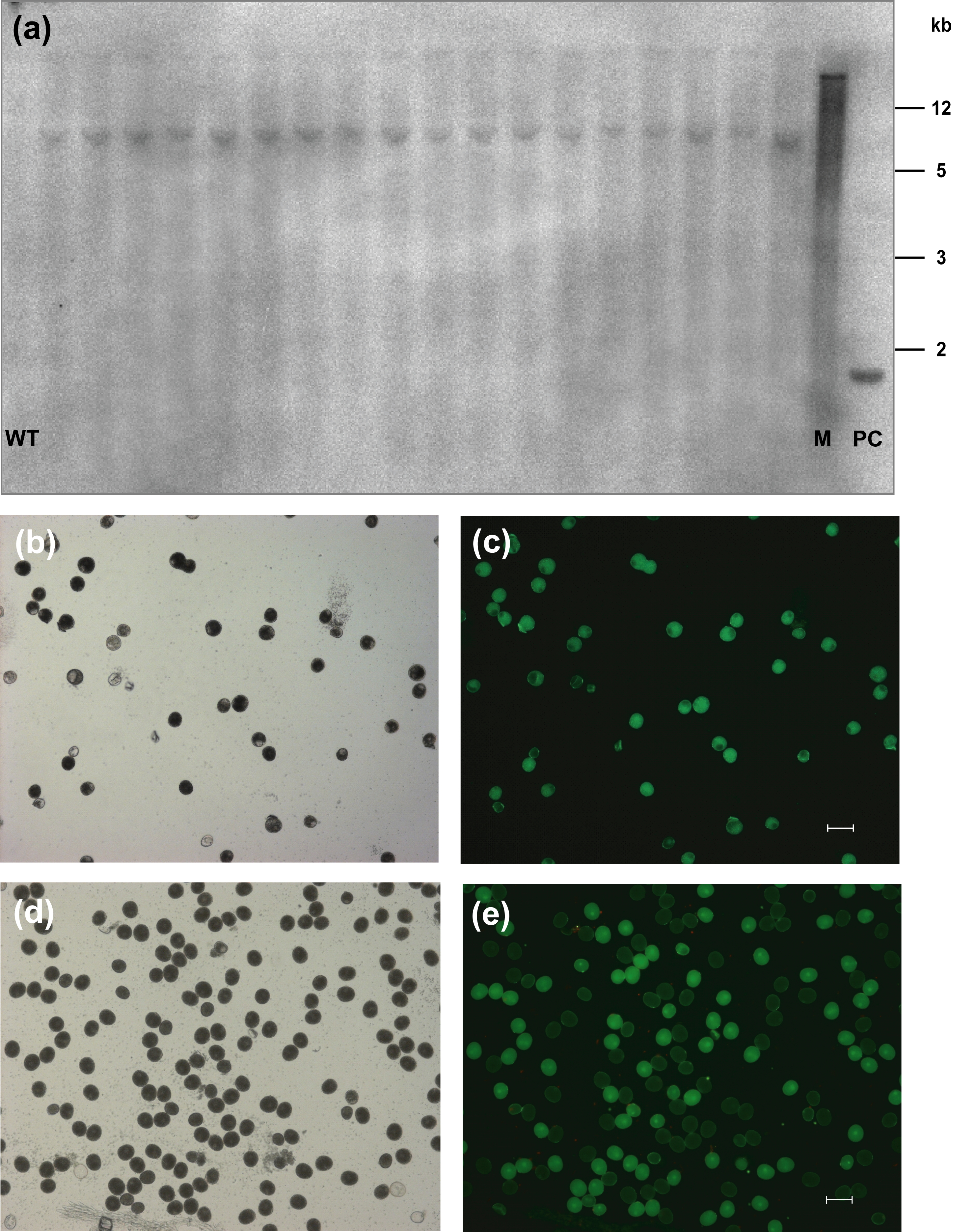


Figure S2. DNA gel blot and *gfp* expression analysis of the donor line PV89. Pollen culture of independent hemizygous transgenic plant is shown as a control. (a) Each T_1_ plant carries a single copy of *gfp*. Genomic DNA digested with *Hin*dIII and hybridized with a *gfp*-specific probe. (c) Immature pollen uniformly expresses *gfp*, while in (e), pollen prepared from an independent hemizygous transgenic (control) plant segregates for *gfp* expression. The same pollen cultures visualized by bright field microscopy are shown on the left (b, d). WT: non-transgenic barley, PC: plasmid control, scale bar = 50 µM.

**24/2-1**

**24/2-2**

**24/2-9**

**24/3-4**

**32/2-2**


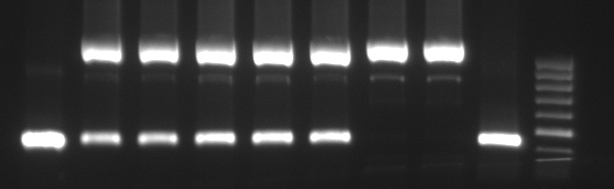


**GFP^+^ barley (donor)**

**barley wild type**

**plasmid (pGH297)**

**plasmid (pGH400)**

**Fok (440bp)**

**Actin (1145bp)**

Figure S3. PCR amplification of the *Fok*I domain from a template of cDNA prepared from each of the primary *gfp* mutants, the *gfp* donor (GFP^+^) and non-transformed cv. ‘Igri’ (wild type). *ACTIN* served as a positive control.

**(a) Heterodimers-BLASTn for GFP-F**

> Target sequence of *gfp* gene

...TGAACCGCATCGAGCTG -(12 nt)- TTCAAGGAGGACGGCAA...

>morex_contig_1694570 CAJW011694570

...TGAACCGCAACGAGCTG -(21 nt)- TTCAAGAGAGATGGATA... 7

>bowman_contig_1398052 CAJX011392892 carma=4HL

...TGAACCTCATCGAGCTG –(14 nt)- TACAACAGCGGCCGAAA... 9

>bowman_contig_913251 CAJX010908226 carma=6HL

...TGACCCGCTTGGAGCTG –(22 nt)- TCAAGAGCGGTGGACAA... 9

>bowman_contig_63271 CAJX010059437 carma=5HL

...TGAATCGCATCGAGCTG –(25 nt)- TTTATGGGGTACTTGTA... 9

>barke_contig_1845717 CAJV011691082 carma=3HS

...TGAACCGCATCGAGCTC –(18 nt)- TGACTGCAGCTCGGTGA... 10

>morex_contig_1575153 CAJW011575153 carma=7HS

...TGAACCGCCTCGAGCTG –(24 nt)- GTACCTGCCGACGACGA... 10

>bowman_contig_143173 CAJX010138923 carma=7HS

...TGAACCGCATCGAACTG -(31 nt)- CTGCATTCGACGGGCAA... 10

>bowman_contig_113158 CAJX010109064 carma=4HS

...TGAACCGCCTCGAGCTG –(12 nt)- CTGATGGGCCGCCTGGA... 12

>morex_contig_185834 CAJW010185834 carma=3HL

...TGAACCGCATCGCGCTG –(19 nt)- CCCCTCGCTCGCCTCGA... 13

>morex_contig_87789 CAJW010087789

...TGAACCGCATCGCGCTG –(19 nt)- CCCCTCGCTCGCCTCGA... 13

**(b) Homodimers-BLASTn for GFP-F**

> Target sequence

...TGAACCGCATCGAGCTG -(12 nt)- CAGCTCGATGCGGTTCA...

>morex_contig_185834 CAJW010185834 carma=3HL

...TGAACCGCATCGCGCTG –(28 nt)- CGCCTCGACCGGCTTCA... 7

>bowman_contig_143173 CAJX010138923 carma=7HS

...TGAACCGCATCGAACTG –(24 nt)- AACTGCACTGCATTCGA... 11

>barke_contig_1845717 CAJV011691082 carma=3HS

...TGAACCGCATCGAGCTC –(21 nt)- CTGCAGCTCGGTGATGA... 11

>morex_contig_123342 CAJW010123342 carma=3HL

...TGAACCTCATCGAGCTG –(17 nt)- CACGGCAGTCGGAAGCA... 11

>morex_contig_1575153 CAJW011575153 carma=7HS

...TGAACCGCCTCGAGCTG –(21 nt)- TACGTACCTGCCGACGA... 11

>morex_contig_58003 CAJW010058003 carma=4HL

...TGGACCGCATCGAGCTG –(30 nt)- CCGCGTATCGCCGCGGA... 11

>bowman_contig_913251 CAJX010908226 carma=6HL

...TGACCCGCTTGGAGCTG –(24 nt) AAGAGCGGTGGACAAAA... 11

>bowman_contig_63271 CAJX010059437 carma=5HL

...TGAATCGCATCGAGCTG –(30 nt)- GGGGTACTTGTAGTAAA... 11

>bowman_contig_1398052 CAJX011392892 carma=4HL

...TGAACCTCATCGAGCTG -(19 nt)- CAGCGGCCGAAAGCGCA... 11

>bowman_contig_113158 CAJX010109064 carma=4HS

...TGAACCGCCTCGAGCTG –(12 nt)- CTGATGGGCCGCCTGGA... 12

**(c) Heterodimers-BLASTn for GFP-R**

> Target sequence of *gfp* gene

...TGAACCGCATCGAGCTG -(12 nt)- TTCAAGGAGGACGGCAA...

>morex_contig_14940 CAJW010014940

...TCGGCGGCGTCGGCCAA –(16 nt)- TTCAAGGAGGACGGCAA... 9

>bowman_contig_1266067 CAJX011260907

...TAACCCGAGTTGCGTCG –(28 nt)- GTCAAGGAGGACGGCAA... 9

> bowman_contig_12217 CAJX010011514 carma=2HS

...TGCGGCGCGTCATCCAC –(27 nt)- CTCAAGGAGGACGGCAA... 9

>bowman_contig_2065182 CAJX012059377

...TGAATAACACCACCAAG –(27 nt)- TTCAAGAAGGACGGCAA... 10

>bowman_contig_1266067 CAJX011260907

...TCGATCGGGAGGAGGCG –(14 nt)- GTCAAGGAGGACGGCAA... 10

>morex_contig_1563877 CAJW011563877 carma=7HS

...TCGACGGCAGGAGGGAC –(21 nt)- GTCAAGGAGGACGGCAA... 11

>bowman_contig_1629214 CAJX011624053

...TGAATAAGACCACCAGT –(27 nt)- TTCAAGGAGGACGGCAA... 11

>bowman_contig_173004 CAJX010168649

...TGGTTAGTGTGGTCCTG –(9 nt)- CGCAAGGAGGACGGCAA... 11

>bowman_contig_68318 CAJX010064484 carma=7HS

...TCGACGGCAGGAGGGAC –(21 nt)- GTCAAGGAGGACGGCAA... 11

>bowman_contig_91300 CAJX010087264 carma=6HS

...TGTAGGACCTTATGATG –(18 nt)- CCCAAGGAGGACGGCAA... 11

**(d) Homodimers-BLASTn for GFP-R**

>Target sequence

...TTGCCGTCCTCCTTGAA -(12 nt)- TTCAAGGAGGACGGCAA...

>morex_contig_14940 CAJW010014940

...TCGGCGGCGTCGGCCAA –(16 nt)- TTCAAGGAGGACGGCAA... 8

>morex_contig_49437 CAJW010049437 carma=6HL

...TCGACGGCCGCAGCAAC –(21 nt)- GTCAAGGAGGACGGCAA... 10

>bowman_contig_895444 CAJX010890549

...TTAGAGCCATCCTCCCA –(27 nt)- GCCAAGGAGGACGGCAA... 10

>bowman_contig_66779 CAJX010062945 carma=6HL

...TCGACGGCCGCAGCAAC –(21 nt)- GTCAAGGAGGACGGCAA... 10

>morex_contig_1563877 CAJW011563877 carma=7HS

...TCGACGGCAGGAGGGAC –(21 nt)- GTCAAGGAGGACGGCAA... 11

>bowman_contig_937263 CAJX010932238

...TGGGTACACTACTGAAA –(20 nt)- GCCAAGGAGGACGGCAA... 11

>bowman_contig_68318 CAJX010064484 carma=7HS

...TCGACGGCAGGAGGGAC –(21 nt)- GTCAAGGAGGACGGCAA... 11

> bowman_contig_12217 CAJX010011514 carma=2HS

...TGCGGCGCGTCATCCAC –(27 nt)- CTCAAGGAGGACGGCAA... 12

>bowman_contig_1266067 CAJX011260907

...TTGCGTCGATCGGGAGG –(19 nt)- GTCAAGGAGGACGGCAA... 12

>bowman_contig_1468620 CAJX011463460

...TGGCCAATGTGATTATG -(9 nt)- CGCAAGGAGGACGGCAA... 12

Figure S4. Best matches found in the barley genome as compared to the target sequence. The target sequences as well as matching nucleotides to the target are highlighted in green, while mismatching nucleotides are indicated by yellow background; numbers at the end represent the mismatches. The spacer nucleotide sequences are replaced by the respective number of nucleotides (nt). Note that optimal spacer length is 10 to 20 nts [12, 26]. (a) The ten best matching contig sequences for left and right TALEN heterodimers, when blast search was performed against the right TALEN binding sequence, (b) the ten best matching contig sequences for homodimers formed from two left TALEN units, (c) the ten best matching contig sequences for left and right TALEN heterodimers, when blast search was performed against the right TALEN binding sequence, (d) the ten best matching contig sequences for homodimers formed from two right TALEN units.


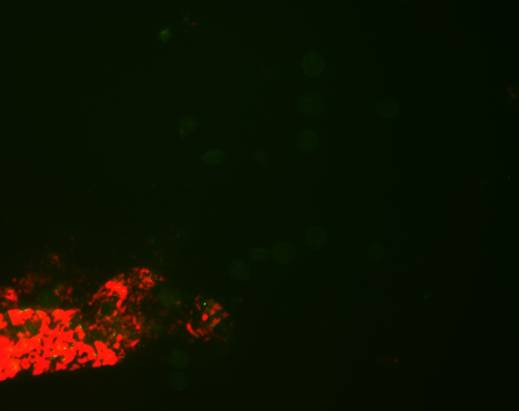

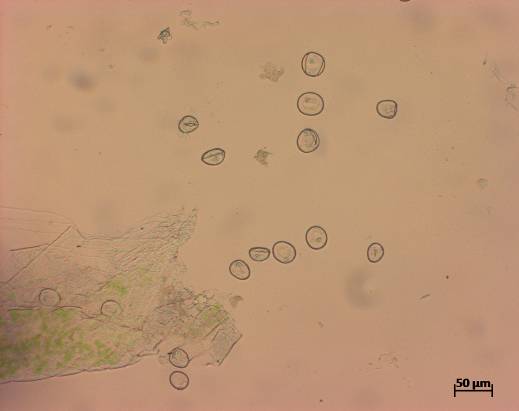

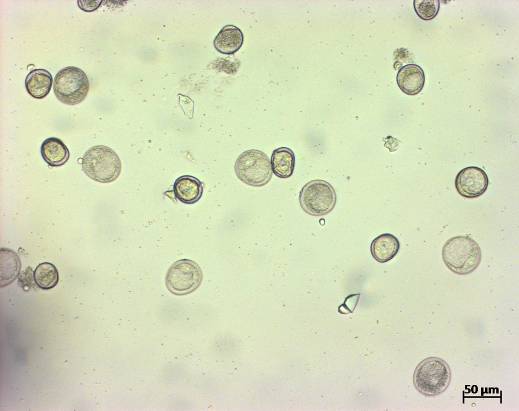

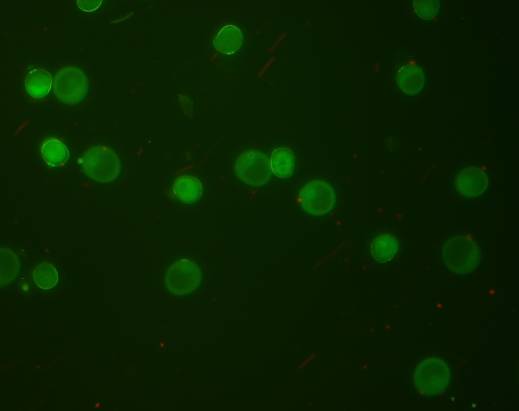


**(a)**

**(b)**

**(c)**

**(d)**

Figure S5. Immature pollen produced by primary transgenic *gfp* knock-out plant 24/2-1 visualized (a) by bright field microscopy, (b) following excitation by far blue light. Chlorophyll autofluorescence from anther tissue appears red. Immature pollen isolated from the *gfp* donor visualized (c) by bright field microscopy, (d) following excitation by far blue light. The latter image reveals accumulation of GFP in pollen, with no segregation being observed.

Table S1. Primer sequences used for the identification of T-DNA elements.

| **Primer** | **Sequence 5’ – 3’** | **Amplified region** |
| --- | --- | --- |
| GH-Bar-F1 | GGTCTGCACCATCGTCAACC | *BAR* gene – forward primer |
| GH-Bar-R1 | TACCGGCAGGCTGAAGTCCA | *BAR* gene – reverse primer |
| FokI-F1 | ATCGAGATCGCCCGGAACAGCACC | *FokI* gene – forward primer |
| FokI-R | ATCATCTCGCCGCCGATCAGGAGC | *FokI* gene – reverse primer |
| ubi-F1 | TTCCGCAGACGGGATCGATCTAGG | TALEN-Left & TALEN-Right units – forward primer |
| TALEN-R2 | TGGCGGCTTGGCGCGTGACAG | TALEN-Left & TALEN-Right units – reverse primer |
| GH-GFP-F1 | GGTCACGAACTCCAGCAGGA | *gfp* gene – reverse primer |
| GH-GFP-R2 | TACGGCAAGCTGACCCTGAA | *gfp* gene – forward primer |

Table S2. Transgenic plants characterized for ploidy level and the presence and expression of TALEN T-DNA.

| **No.** | **Transgenic identifier** | **FokI** | **BAR** | **TALEN-Left** | **TALEN-Right** | **FokI expression** | **Ploidy** |
| --- | --- | --- | --- | --- | --- | --- | --- |
| 1 | 24/2 -1 | + | + | + | - | + | 1n |
| 2 | 24/2 -2 | + | + | + | - | + | 2n |
| 3 | 24/2 -3 | + | + | + | + | + | 1n |
| 4 | 24/2 -4 | + | + | - | + | + | 1n |
| 5 | 24/2 -5 | + | + | + | - | + | 1n |
| 6 | 24/2 -6 | + | + | + | - | + | 1n/2n |
| 7 | 24/2 -7 | + | + | - | + | + | 3n |
| 8 | 24/2 -8 | + | + | + | - | + | 2n |
| 9 | 24/2 -9 | + | + | + | - | + | 1n |
| 10 | 24/3 -1 | + | + | + | - | + | 1n |
| 11 | 24/3 -2 | + | + | + | - | + | 1n |
| 12 | 24/3 -3 | + | + | + | - | + | 1n |
| 13 | 24/3 -4 | + | + | + | - | + | 1n/2n |
| 14 | 24/3 -5 | + | + | + | - | + | 2n |
| 15 | 32/2 -2 | + | + | + | + | + | 2n |
| 16 | 32/2 -3 | + | + | + | - | + | 1n |
| 17 | 32/2 -5 | + | + | + | - | + | 1n |
| 18 | 32/2 -6 | + | + | + | - | + | 1n/2n |

Sequences of the used binary vectors:

**>pGH400 (7d35S-Ubi-NLS-HA-TAL-Right)**

TCTAGAACTAGTGGATCCCCCGGGCTGCAGGAATTCGATCTATGTTACTAGATCGGGCCAACATGGTGGAGCACGACACTCTCGTCTACTCCAAGAATATCAAAGATACAGTCTCAGAAGACCAAAGGGCTATTGAGACTTTTCAACAAAGGGTAATATCGGGAAACCTCCTCGGATTCCATTGCCCAGCTATCTGTCACTTCATCAAAAGGACAGTAGAAAAGGAAGGTGGCACCTACAAATGCCATCATTGCGATAAAGGAAAGGCTATCGTTCAAGATGCCTCTGCCGACAGTGGTCCCAAAGATGGACCCCCACCCACGAGGAGCATCGTGGAAAAAGAAGACGTTCCAACCACGTCTTCAAAGCAAGTGGATTGATGTGATAACATGGTGGAGCACGACACTCTCGTCTACTCCAAGAATATCAAAGATACAGTCTCAGAAGACCAAAGGGCTATTGAGACTTTTCAACAAAGGGTAATATCGGGAAACCTCCTCGGATTCCATTGCCCAGCTATCTGTCACTTCATCAAAAGGACAGTAGAAAAGGAAGGTGGCACCTACAAATGCCATCATTGCGATAAAGGAAAGGCTATCGTTCAAGATGCCTCTGCCGACAGTGGTCCCAAAGATGGACCCCCACCCACGAGGAGCATCGTGGAAAAAGAAGACGTTCCAACCACGTCTTCAAAGCAAGTGGATTGATGTGATATCTCCACTGACGTAAGGGATGACGCACAATCCCACTATCCTTCGCAAGACCTTCCTCTATATAAGGAAGTTCATTTCATTTGGAGAGGACACGCTGAAATCACCAGTCTCTCTCTACAAATCTATCTCTCTCGAGTGGCCACCATGGGCCCAGAACGACGCCCGGCCGACATCCGCCGTGCCACCGAGGCGGACATGCCGGCGGTCTGCACCATCGTCAACCACTACATCGAGACAAGCACGGTCAACTTCCGTACCGAGCCGCAGGAACCGCAGGAGTGGACGGACGACCTCGTCCGTCTGCGGGAGCGCTATCCCTGGCTCGTCGCCGAGGTGGACGGCGAGGTCGCCGGCATCGCCTACGCGGGCCCCTGGAAGGCACGCAACGCCTACGACTGGACGGCCGAGTCGACCGTGTACGTCTCCCCCCGCCACCAGCGGACGGGACTGGGCTCCACGCTCTACACCCACCTGCTGAAGTCCCTGGAGGCACAGGGCTTCAAGAGCGTGGTCGCTGTCATCGGGCTGCCCAACGACCCGAGCGTGCGCATGCACGAGGCGCTCGGATATGCCCCCCGCGGCATGCTGCGGGCGGCCGGCTTCAAGCACGGGAACTGGCATGACGTGGGTTTCTGGCAGCTGGACTTCAGCCTGCCGGTACCGCCCCGTCCGGTCCTGCCCGTCACCGAGATCTGAGATCACGCGTTCTAGTCCGCAAAAATCACCAGTCTCTCTCTACAAATCTATCTCTCTCTATTTTTCTCCAGAATAATGTGTGAGTAGTTCCCAGATAAGGGAATTAGGGTTCTTATAGGGTTTCGCTCATGTGTTGAGCATATAAGAAACCCTTAGTATGTATTTGTATTTGTAAAATACTTCTATCAATAAAATTTCTAATTCCTAAAACCAAAATCCAGTGACCTGCAGGCATGCAAGCTGATCCACTAGAGGCCATGGCGGCCGCGTCGAGCGATCTAGTAACATAGATGACACCGCGCGCGATAATTTATCCTAGTTTGCGCGCTATATTTTGTTTTCTATCGCGTATTAAATGTATAATTGCGGGACTCTAATCATAAAAACCCATCTCATAAATAACGTCATGCATTACATGTTAATTATTACATGCTTAACGTAATTCAACAGAAATTATATGATAATCATCGCAAGACCGGCAACAGGATTCAATCTTAAGAAACTTTATTGCCAAATGTTTGAACGATCGGGGAAATTCGAGTCGACACGCGTAAGCTTCAGCTGCTGCAGGCTCGAGGAGCTCGTCTAGAGGATCGCTCGAGTTATCAGTCGGCCGCGAAGTTGATCTCGCCGTTGTTGAACTTCCTCCTCACCTCCTCCAGGGTCAGGGTGCCGGCCTTGATCATCTCGCCGCCGATCAGGAGCTCCTCCACGGACAGCACGGCGCCGTTGCAGTTGGTGATGTGGTTCAGCCTGGTCAGCTGGGCCTTGTAGTTGCCCTTGAAGTGGCCGGACACGAACAGGAACTTGAACTCGGTCACGCTGGAGGGGTACACCTTCCACCACTCGTTGGGGTTGATGTGCTTGTTCCTGGTCTGGTTCTCCTCCACGTACCTCTGCATTTCGTCGGCCTGGCCGATGGGCAGGTTGTAGCCGCCGGAGTAGGCCTTGGTGTCCACGATCACGCCGTAGTCGATGGGGGAGCCCACGGTGTAGATGGCGCCGTCGGGCTTCCTGGAGCCGCCCAGGTGCTTGCCCCTGTAGCCGTACACCTTCATGAAGAACTCCATCACCTTCATCTCCAGGATACGGTCCTGGGTGCTGTTCCGGGCGATCTCGATCAGCTCGATGTACTCGTGGGGCACGTACTTCAGCTTGTGCCTCAACTCGGATTTCTTCTCCTCCAGCTCGGACTTCACCAGCTGGGAACGGCTGATAGGATCCCCGCCACTTCCGCTGCCACTCGGATCAGGGCGAGATAACTGGGCAACAATGCTCTCCAGCGCCGGCCTGCCGCCGCCATTGCTGGCGATGGCCACCACCTGCTGAGGGGTCAAGCCGTGGGCCTGGCACAGCACCGGCAACAGCGCCTGCACCGTCTCCAGCGCCTGCTTGCCACCAATATTGCTGGCGATGGCCACCACCTGCTCCGGGGTCAAGCCGTGGGCCTGGCACAGCACCGGCAACAGCCGCTGGACCGTCTCCAGCGCCTGCTTGCCACCATTATTGCTGGCGATGGCCACCACCTGCTGGGGGGTCAAGCCGTGGGCCTGGCACAGCACCGGCAACAGCCGCTGGACCGTCTCCAGCGCCTGCTTGCCACCGCCATTGCTGGCGATGGCCACCACCTGCTGGGGGGTCAAGCCGTGGGCCTGGCACAGCACCGGCAACAGCCGCTGGACCGTCTCCAGCGCCTGCTTGCCACCGCCATTGCTGGCGATGGCCACCACCTGCTGGGGGGTCAAGCCGTGGGCCTGGCACAGCACCGGCAACAGCCGCTGGACCGTCTCCAGCGCCTGCTTGCCGCCATCGTGGCTGGCGATGGCCACCACCTGCTCCGGGGTCAAGCCGTGGGCCTGGCACAGCACCGGCAACAGCCGCTGGACCGTCTCCAGCGCCTGCTTGCCGCCATCGTGGCTGGCGATGGCCACCACCTGCTCCGGGGTCAAGCCGTGGGCCTGGCACAGCACCGGCAACAGCCGCTGGACCGTCTCCAGCGCCTGCTTGCCACCGCCATTGCTGGCGATGGCCACCACCTGCTGGGGGGTCAAGCCGTGGGCCTGGCACAGCACCGGCAACAGCCGCTGGACCGTCTCCAGCGCCTGCTTGCCGCCATCGTGGCTGGCGATGGCCACCACCTGCTCCGGGGTCAAGCCGTGGGCCTGGCACAGCACCGGCAACAGCCGCTGGACCGTCTCCAGCGCCTGCTTGCCGCCATCGTGGCTGGCGATGGCCACCACCTGCTCCGGGGTCAAGCCGTGGGCCTGGCACAGCACCGGCAACAGCCGCTGGACCGTCTCCAGCGCCTGCTTGCCACCGCCATTGCTGGCGATGGCCACCACCTGCTGGGGGGTCAAGCCGTGGGCCTGGCACAGCACCGGCAACAGCCGCTGGACCGTCTCCAGCGCCTGCTTGCCACCATTATTGCTGGCGATGGCCACCACCTGCTGGGGGGTCAAGCCGTGGGCCTGGCACAGCACCGGCAACAGCCGCTGGACCGTCTCCAGCGCCTGCTTGCCGCCATCGTGGCTGGCGATGGCCACCACCTGCTCCGGGGTCAAGCCGTGGGCCTGGCACAGCACCGGCAACAGCCGCTGGACCGTCTCCAGCGCCTGCTTGCCGCCATCGTGGCTGGCGATGGCCACCACCTGCTCCGGGGTCAAGCCGTGGGCCTGGCACAGCACCGGCAACAGCCGCTGGACCGTCTCCAGCGCCTGCTTGCCACCATTATTGCTGGCGATGGCCACCACCTGCTGGGGGGTCAAGCCGTGGGCCTGGCACAGCACCGGCAACAGCCGCTGGACCGTCTCCAGCGCCTGCTTGCCACCGCCATTGCTGGCGATGGCCACCACCTGCTGGGGGGTCAAGTTGAGCGGGGCACCCGTCAGTGCATTGCGCCATGCATGCACTGCCTCCACTGCGGTCACGCCGCCACGTTTTGCAATCTTGAGAAGTTGGCCTGTGTCCAACTGTAACGGTGGACCTCTCAACTCTCCCGCCACCGTGAGCAAGGCCTCCAGAGCGCGTGCGCCGGACCACTGTTTGCCGACGCCAACGATCGCTTCGTGTGTCGCCTCTGGCAACGCTGCGATCATGTCCTGATACTTGACAGCGACGGTCCCTAACGCTGCCGGGTGTTGGCTTAACGCAACGATGTGCGCGTGTGTAAACCCGTGGCCGACCAGTGCCTCGTGGTGCTGCGCCACTGTCGAACGAACCTTCGGTTTGATCTTCTCCTGTTGCTGCTGGCTGTAGCCGAGCGTGCGTAGATCCACCTGCGCCGCCGGCGAAGCGTCGGAGGGTTGCGCAGCACGTCGTCGCGGCGCCGGCTTGGCGCGCGGGGGCCGCGCGGCAGTGACAGCCACGCGCATGGTGGGTGGGGGGGCGTCGGCTGCCCGCAGACCCGATTGCACCTCATCCCACTCGCCTGTGGCAGCCTCTGTATGGTGAGCGCCGAAGGGAGGCAATGAATCAAAAAGCGATGTATTAAAAAGTGACGGATCGAACTGACGTAACAGGTCACTGAAGCTGCCCGCCGAGAACGCAGGTGAGGGGGCAGGGGGAGATGGCAGCCGGGTCCGGGACATCGTCCGCCGAGCCGGCAAGCCATCCAGGGGGCCGCCGGCAGGCGGAGACACCCCACGATCTGCAGTCGGCTGAACCCCATCGGGTTGGGGTCCGGGCAGAAGCTCGCGGGCAGGACTTGGTGTGCGCGAACGAATGGGGTCGGCCATGGTGGCGGCTTGGCGCGTGACAGCGCTAGCGTAGTCTGGGACGTCGTATGGGTAGACCTTTCTCTTCTTCTTTGGAGCCATGGCGCGCCGAACTAGTACCTGCAGAAGTAACACCAAACAACAGGGTGAGCATCGACAAAAGAAACAGTACCAAGCAAATAAATAGCGTATGAAGGCAGGGCTAAAAAAATCCACATATAGCTGCTGCATATGCCATCATCCAAGTATATCAAGATCAAAATAATTATAAAACATACTTGTTTATTATAATAGATAGGTACTCAAGGTTAGAGCATATGAATAGATGCTGCATATGCCATCATGTATATGCATCAGTAAAACCCACATCAACATGTATACCTATCCTAGATCGATCCCGTCTGCGGAACGGCTAGAGCCATCCCAGGATTCCCCAAAGAGAAACACTGGCAAGTTAGCAATCAGAACGTGTCTGACGTACAGGTCGCATCCGTGTACGAACGCTAGCAGCACGGATCTAACACAAACACGGATCTAACACAAACATGAACAGAAGTAGAACTACCGGGCCCTAACCATGGACCGGAACGCCGATCTAGAGAAGGTAGAGAGGGGGGGGGGGGGAGGACGAGCGGCGTACCTTGAAGCGGAGGTGCCGACGGGTGGATTTGGGGGAGATCTGGTTGTGTGTGTGTGCGCTCCGAACAACACGAGGTTGGGGAAAGAGGGTGTGGAGGGGGTGTCTATTTATTACGGCGGGCGAGGAAGGGAAAGCGAAGGAGCGGTGGGAAAGGAATCCCCCGTAGCTGCCGGTGCCGTGAGAGGAGGAGGAGGCCGCCTGCCGTGCCGGCTCACGTCTGCCGCTCCGCCACGCAATTTCTGGATGCCGACAGCGGAGCAAGTCCAACGGTGGAGCGGAACTCTCGAGAGGGGTCCAGAGGCAGCGACAGAGATGCCGTGCCGTCTGCTTCGCTTGGCCCGACGCGACGCTGCTGGTTCGCTGGTTGGTGTCCGTTAGACTCGTCGATCGACGGCGTTTAACAGGCTGGCATTATCTACTCGAAACAAGAAAAATGTTTCCTTAGTTTTTTTAATTTCTTAAAGGGTATTTGTTTAATTTTTAGTCACTTTATTTTATTCTATTTTATATCTAAATTATTAAATAAAAAAACTAAAATAGAGTTTTAGTTTTCTTAATTTAGAGGCTAAAATAGAATAAAATAGATGTACTAAAAAAATTAGTCTATAAAAACCATTAACCCTAAACCCTAAATGGATGTACTAATAAAATGGATGAAGTATTATATAGGTGAAGCTATTTGCAAAAAAAAAGGAGAACACATGCACACTAAAAAGATAAAACTGTAGAGTCCTGTTGTCAAAATACTCAATTGTCCTTTAGACCATGTCTAACTGTTCATTTATATGATTCTCTAAAACACTGATATTATTGTAGTACTATAGATTATATTATTCGTAGAGTAAAGTTTAAATATATGTATAAAGATAGATAAACTGCACTTCAAACAAGTGTGACAAAAAAAATATGTGGTAATTTTTTATAACTTAGACATGCAATGCTCATTATCTCTAGAGAGGGGCACGACCGGGTCACGCTGCACTGCAGCCTAGTAAGGCCTTAAGGGCCAGATCTTGGGCCCGGTACCCGATCAGATTGTCGTTTCCCGCCTTCGGTTTAAACTATCAGTGTTTGACAGGATATATTGGCGGGTAAACCTAAGAGAAAAGAGCGTTTATTAGAATAATCGGATATTTAAAAGGGCGTGAAAAGGTTTATCCGTTCGTCCATTTGTATGTGCATGCCAACCACAGGGTTCCCCTCGGGAGTGCTTGGCATTCCGTGCGATAATGACTTCTGTTCAACCACCCAAACGTCGGAAAGCCTGACGACGGAGCAGCATTCCAAAAAGATCCCTTGGCTCGTCTGGGTCGGCTAGAAGGTCGAGTGGGCTGCTGTGGCTTGATCCCTCAACGCGGTCGCGGACGTAGCGCAGCGCCGAAAAATCCTCGATCGCAAATCCGACGCTGTCGAAAAGCGTGATCTGCTTGTCGCTCTTTCGGCCGACGTCCTGGCCAGTCATCACGCGCCAAAGTTCCGTCACAGGATGATCTGGCGCGAGTTGCTGGATCTCGCCTTCAATCCGGGTCTGTGGCGGGAACTCCACGAAAATATCCGAACGCAGCAAGATATCGCGGTGCATCTCGGTCTTGCCTGGGCAGTCGCCGCCGACGCCGTTGATGTGGACGCCGAAAAGGATCTAGGTGAAGATCCTTTTTGATAATCTCATGACCAAAATCCCTTAACGTGAGTTTTCGTTCCACTGAGCGTCAGACCCCGTAGAAAAGATCAAAGGATCTTCTTGAGATCCTTTTTTTCTGCGCGTAATCTGCTGCTTGCAAACAAAAAAACCACCGCTACCAGCGGTGGTTTGTTTGCCGGATCAAGAGCTACCAACTCTTTTTCCGAAGGTAACTGGCTTCAGCAGAGCGCAGATACCAAATACTGTTCTTCTAGTGTAGCCGTAGTTAGGCCACCACTTCAAGAACTCTGTAGCACCGCCTACATACCTCGCTCTGCTAATCCTGTTACCAGTGGCTGCTGCCAGTGGCGATAAGTCGTGTCTTACCGGGTTGGACTCAAGACGATAGTTACCGGATAAGGCGCAGCGGTCGGGCTGAACGGGGGGTTCGTGCACACAGCCCAGCTTGGAGCGAACGACCTACACCGAACTGAGATACCTACAGCGTGAGCTATGAGAAAGCGCCACGCTTCCCGAAGGGAGAAAGGCGGACAGGTATCCGGTAAGCGGCAGGGTCGGAACAGGAGAGCGCACGAGGGAGCTTCCAGGGGGAAACGCCTGGTATCTTTATAGTCCTGTCGGGTTTCGCCACCTCTGACTTGAGCGTCGATTTTTGTGATGCTCGTCAGGGGGGCGGAGCCTATGGAAAAACGCCAGCAACGCGGCCTTTTTACGGTTCCTGGCCTTTTGCTGGCCTTTTGCTCACATGTTCTTTCCTGCGTTATCCCCTGATTCTGTGGATAACCGATTACCGCCTTTGAGTGAGCTGATACCGCTCGCCGCAGCCGAACGACCGAGCGCAGCGAGTCAGTGAGCGAGGAAGCGGAAGAGCGCCTGATGCGGTATTTTCTCCTTACGCATCTGTGCGGTATTTCACACCGCATATGGTGCACTCTCAGTACAATCTGCTCTGATGCCGCATAGTTAAGCCAGTATACACTCCGCTATCGCTACGTGACTGGGTCATGGCTGCGCCCCGACACCCGCCAACACCCGCTGACGCGCCCTGACGGGCTTGTCTGCTCCCGGCATCCGCTTACAGACAAGCTGTGACCGTCTCCGGGAGCTGCATGTGTCAGAGGTTTTCACCGTCATCACCGAAACGCGCGAGGCAGGGGTACGTCGAGGTCGATCCAACCCCTCCGCTGCTATAGTGCAGTCGGCTTCTGACGTTCAGTGCAGCCGTCTTCTGAAAACGACATGTCGCACAAGTCCTAAGTTACGCGACAGGCTGCCGCCCTGCCCTTTTCCTGGCGTTTTCTTGTCGCGTGTTTTAGTCGCATAAAGTAGAATACTTGCGACTAGAACCGGAGACATTACGCCATGAACAAGAGCGCCGCCGCTGGCCTGCTGGGCTATGCCCGCGTCAGCACCGACGACCAGGACTTGACCAACCAACGGGCCGAACTGCACGCGGCCGGCTGCACCAAGCTGTTTTCCGAGAAGATCACCGGCACCAGGCGCGACCGCCCGGAGCTGGCCAGGATGCTTGACCACCTACGCCCTGGCGACGTTGTGACAGTGACCAGGCTAGACCGCCTGGCCCGCAGCACCCGCGACCTACTGGACATTGCCGAGCGCATCCAGGAGGCCGGCGCGGGCCTGCGTAGCCTGGCAGAGCCGTGGGCCGACACCACCACGCCGGCCGGCCGCATGGTGTTGACCGTGTTCGCCGGCATTGCCGAGTTCGAGCGTTCCCTAATCATCGACCGCACCCGGAGCGGGCGCGAGGCCGCCAAGGCGCGAGGCGTGAAGTTTGGCCCCCGCCCTACCCTCACCCCGGCACAGATCGCGCACGCCCGCGAGCTGATCGACCAGGAAGGCCGCACCGTGAAAGAGGCGGCTGCACTGCTTGGCGTGCATCGCTCGACCCTGTACCGCGCACTTGAGCGCAGCGAGGAAGTGACGCCCACCGAGGCCAGGCGGCGCGGTGCCTTCCGTGAGGACGCATTGACCGAGGCCGACGCCCTGGCGGCCGCCGAGAATGAACGCCAAGAGGAACAAGCATGAAACCGCACCAGGACGGCCAGGACGAACCGTTTTTCATTACCGAAGAGATCGAGGCGGAGATGATCGCGGCCGGGTACGTGTTCGAGCCGCCCGCGCACGTCTCAACCGTGCGGCTGCATGAAATCCTGGCCGGTTTGTCTGATGCCAAGCTCGCGGCCTGGCCGGCGAGCTTGGCCGCTGAAGAAACCGAGCGCCGCCGTCTAAAAAGGTGATGTGTATTTGAGTAAAACAGCTTGCGTCATGCGGTCGCTGCGTATATGATGCGATGAGTAAATAAACAAATACGCAAGGGGAACGCATGAAGGTTATCGCTGTACTTAACCAGAAAGGCGGGTCAGGCAAGACGACCATCGCAACCCATCTAGCCCGCGCCCTGCAACTCGCCGGGGCCGATGTTCTGTTAGTCGATTCCGATCCCCAGGGCAGTGCCCGCGATTGGGCGGCCGTGCGGGAAGATCAACCGCTAACCGTTGTCGGCATCGACCGCCCGACGATTGACCGCGACGTGAAGGCCATCGGCCGGCGCGACTTCGTAGTGATCGACGGAGCGCCCCAGGCGGCGGACTTGGCTGTGTCCGCGATCAAGGCAGCCGACTTCGTGCTGATTCCGGTGCAGCCAAGCCCTTACGACATATGGGCCACCGCCGACCTGGTGGAGCTGGTTAAGCAGCGCATTGAGGTCACGGATGGAAGGCTACAAGCGGCCTTTGTCGTGTCGCGGGCGATCAAAGGCACGCGCATCGGCGGTGAGGTTGCCGAGGCGCTGGCCGGGTACGAGCTGCCCATTCTTGAGTCCCGTATCACGCAGCGCGTGAGCTACCCAGGCACTGCCGCCGCCGGCACAACCGTTCTTGAATCAGAACCCGAGGGCGACGCTGCCCGCGAGGTCCAGGCGCTGGCCGCTGAAATTAAATCAAAACTCATTTGAGTTAATGAGGTAAAGAGAAAATGAGCAAAAGCACAAACACGCTAAGTGCCGGCCGTCCGAGCGCACGCAGCAGCAAGGCTGCAACGTTGGCCAGCCTGGCAGACACGCCAGCCATGAAGCGGGTCAACTTTCAGTTGCCGGCGGAGGATCACACCAAGCTGAAGATGTACGCGGTACGCCAAGGCAAGACCATTACCGAGCTGCTATCTGAATACATCGCGCAGCTACCAGAGTAAATGAGCAAATGAATAAATGAGTAGATGAATTTTAGCGGCTAAAGGAGGCGGCATGGAAAATCAAGAACAACCAGGCACCGACGCCGTGGAATGCCCCATGTGTGGAGGAACGGGCGGTTGGCCAGGCGTAAGCGGCTGGGTTGTCTGCCGGCCCTGCAATGGCACTGGAACCCCCAAGCCCGAGGAATCGGCGTGAGCGGTCGCAAACCATCCGGCCCGGTACAAATCGGCGCGGCGCTGGGTGATGACCTGGTGGAGAAGTTGAAGGCGGCGCAGGCCGCCCAGCGGCAACGCATCGAGGCAGAAGCACGCCCCGGTGAATCGTGGCAAGCGGCCGCTGATCGAATCCGCAAAGAATCCCGGCAACCGCCGGCAGCCGGTGCGCCGTCGATTAGGAAGCCGCCCAAGGGCGACGAGCAACCAGATTTTTTCGTTCCGATGCTCTATGACGTGGGCACCCGCGATAGTCGCAGCATCATGGACGTGGCCGTTTTCCGTCTGTCGAAGCGTGACCGACGAGCTGGCGAGGTGATCCGCTACGAGCTTCCAGACGGGCACGTAGAGGTTTCCGCAGGGCCGGCCGGCATGGCGAGTGTGTGGGATTACGACCTGGTACTGATGGCGGTTTCCCATCTAACCGAATCCATGAACCGATACCGGGAAGGGAAGGGAGACAAGCCCGGCCGCGTGTTCCGTCCACACGTTGCGGACGTACTCAAGTTCTGCCGGCGAGCCGATGGCGGAAAGCAGAAAGACGACCTGGTAGAAACCTGCATTCGGTTAAACACCACGCACGTTGCCATGCAGCGTACGAAGAAGGCCAAGAACGGCCGCCTGGTGACGGTATCCGAGGGTGAAGCCTTGATTAGCCGCTACAAGATCGTAAAGAGCGAAACCGGGCGGCCGGAGTACATCGAGATCGAGCTAGCTGATTGGATGTACCGCGAGATCACAGAAGGCAAGAACCCGGACGTGCTGACGGTTCACCCCGATTACTTTTTGATCGATCCCGGCATCGGCCGTTTTCTCTACCGCCTGGCACGCCGCGCCGCAGGCAAGGCAGAAGCCAGATGGTTGTTCAAGACGATCTACGAACGCAGTGGCAGCGCCGGAGAGTTCAAGAAGTTCTGTTTCACCGTGCGCAAGCTGATCGGGTCAAATGACCTGCCGGAGTACGATTTGAAGGAGGAGGCGGGGCAGGCTGGCCCGATCCTAGTCATGCGCTACCGCAACCTGATCGAGGGCGAAGCATCCGCCGGTTCCTAATGTACGGAGCAGATGCTAGGGCAAATTGCCCTAGCAGGGGAAAAAGGTCGAAAAGGTCTCTTTCCTGTGGATAGCACGTACATTGGGAACCCAAAGCCGTACATTGGGAACCGGAACCCGTACATTGGGAACCCAAAGCCGTACATTGGGAACCGGTCACACATGTAAGTGACTGATATAAAAGAGAAAAAAGGCGATTTTTCCGCCTAAAACTCTTTAAAACTTATTAAAACTCTTAAAACCCGCCTGGCCTGTGCATAACTGTCTGGCCAGCGCACAGCCGAAGAGCTGCAAAAAGCGCCTACCCTTCGGTCGCTGCGCTCCCTACGCCCCGCCGCTTCGCGTCGGCCTATCGCGGCCGCTGGCCGCTCAAAAATGGCTGGCCTACGGCCAGGCAATCTACCAGGGCGCGGACAAGCCGCGCCGTCGCCACTCGACCGCCGGCGCCCACATCAAGGCACCGGTGGGTATGCCTGACGATGCGTGGAGACCGAAACCTTGCGCTCGTTCGCCAGCCAGGACAGAAATGCCTCGACTTCGCTGCTGCCCAAGGTTGCCGGGTGACGCACACCGTGGAAACGGATGAAGGCACGAACCCAGTGGACATAAGCCTGTTCGGTTCGTAAGCTGTAATGCAAGTAGCGTATGCGCTCACGCAACTGGTCCAGAACCTTGACCGAACGCAGCGGTGGTAACGGCGCAGTGGCGGTTTTCATGGCTTGTTATGACTGTTTTTTTGGGGTACAGTCTATGCCTCGGGCATCCAAGCAGCAAGCGCGTTACGCCGTGGGTCGATGTTTGATGTTATGGAGCAGCAACGATGTTACGCAGCAGGGCAGTCGCCCTAAAACAAAGTTAAACATCATGAGGGAAGCGGTGATCGCCGAAGTATCGACTCAACTATCAGAGGTAGTTGGCGTCATCGAGCGCCATCTCGAACCGACGTTGCTGGCCGTACATTTGTACGGCTCCGCAGTGGATGGCGGCCTGAAGCCACACAGTGATATTGATTTGCTGGTTACGGTGACCGTAAGGCTTGATGAAACAACGCGGCGAGCTTTGATCAACGACCTTTTGGAAACTTCGGCTTCCCCTGGAGAGAGCGAGATTCTCCGCGCTGTAGAAGTCACCATTGTTGTGCACGACGACATCATTCCGTGGCGTTATCCAGCTAAGCGCGAACTGCAATTTGGAGAATGGCAGCGCAATGACATTCTTGCAGGTATCTTCGAGCCAGCCACGATCGACATTGATCTGGCTATCTTGCTGACAAAAGCAAGAGAACATAGCGTTGCCTTGGTAGGTCCAGCGGCGGAGGAACTCTTTGATCCGGTTCCTGAACAGGATCTATTTGAGGCGCTAAATGAAACCTTAACGCTATGGAACTCGCCGCCCGACTGGGCTGGCGATGAGCGAAATGTAGTGCTTACGTTGTCCCGCATTTGGTACAGCGCAGTAACCGGCAAAATCGCGCCGAAGGATGTCGCTGCCGACTGGGCAATGGAGCGCCTGCCGGCCCAGTATCAGCCCGTCATACTTGAAGCTAGACAGGCTTATCTTGGACAAGAAGAAGATCGCTTGGCCTCGCGCGCAGATCAGTTGGAAGAATTTGTCCACTACGTGAAAGGCGAGATCACCAAGGTAGTCGGCAAATAATGTCTAACAATTCGTTCAAGCCGACGCCGCTTCGCGGCGCGGCTTAACTCAAGCGTTAGATGCACTAAGCACATAATTGCTCACAGCCAAACTATCAGGTCAAGTCTGCTTTTATTATTTTTAAGCGTGCATAATAAGCCCTACACAAATTGGGAGATATATCATGAAAGGCTGGCTTTTTCTTGTTATCGCAATAGTTGGCGAAGTAATCGCAACATAGCTTGCTTGGTCGTTCCGCGTGAACGTCGGCTCGATTGTACCTGCGTTCAAATACTTTGCGATCGTGTTGCGCGCCTGCCCGGTGCGTCGGCTGATCTCACGGATCGACTGCTTCTCTCGCAACGCCATCCGACGGATGATGTTTAAAAGTCCCATGTGGATCACTCCGTTGCCCCGTCGCTCACCGTGTTGGGGGGAAGGTGCACATGGCTCAGTTCTCAATGGAAATTATCTGCCTAACCGGCTCAGTTCTGCGTAGAAACCAACATGCAAGCTCCACCGGGTGCAAAGCGGCAGCGGCGGCAGGATATATTCAATTGTAAATGGCTTCATGTCCGGGAAATCTACATGGATCAGCAATGAGTATGATGGTCAATATGGAGAAAAAGAAAGAGTAATTACCAATTTTTTTTCAATTCAAAAATGTAGATGTCCGCAGCGTTATTATAAAATGAAAGTACATTTTGATAAAACGACAAATTACGATCCGTCGTATTTATAGGCGAAAGCAATAAACAAATTATTCTAATTCGGAAATCTTTATTTCGACGTGTCTACATTCACGTCCAAATGGGGGCTTAGATGAGAAACTTCACGATCGGC

**>pGH297 (p7d35S-Ubi-NLS-TAL-left)**

TCTAGAACTAGTGGATCCCCCGGGCTGCAGGAATTCGATCTATGTTACTAGATCGGGCCAACATGGTGGAGCACGACACTCTCGTCTACTCCAAGAATATCAAAGATACAGTCTCAGAAGACCAAAGGGCTATTGAGACTTTTCAACAAAGGGTAATATCGGGAAACCTCCTCGGATTCCATTGCCCAGCTATCTGTCACTTCATCAAAAGGACAGTAGAAAAGGAAGGTGGCACCTACAAATGCCATCATTGCGATAAAGGAAAGGCTATCGTTCAAGATGCCTCTGCCGACAGTGGTCCCAAAGATGGACCCCCACCCACGAGGAGCATCGTGGAAAAAGAAGACGTTCCAACCACGTCTTCAAAGCAAGTGGATTGATGTGATAACATGGTGGAGCACGACACTCTCGTCTACTCCAAGAATATCAAAGATACAGTCTCAGAAGACCAAAGGGCTATTGAGACTTTTCAACAAAGGGTAATATCGGGAAACCTCCTCGGATTCCATTGCCCAGCTATCTGTCACTTCATCAAAAGGACAGTAGAAAAGGAAGGTGGCACCTACAAATGCCATCATTGCGATAAAGGAAAGGCTATCGTTCAAGATGCCTCTGCCGACAGTGGTCCCAAAGATGGACCCCCACCCACGAGGAGCATCGTGGAAAAAGAAGACGTTCCAACCACGTCTTCAAAGCAAGTGGATTGATGTGATATCTCCACTGACGTAAGGGATGACGCACAATCCCACTATCCTTCGCAAGACCTTCCTCTATATAAGGAAGTTCATTTCATTTGGAGAGGACACGCTGAAATCACCAGTCTCTCTCTACAAATCTATCTCTCTCGAGTGGCCACCATGGGCCCAGAACGACGCCCGGCCGACATCCGCCGTGCCACCGAGGCGGACATGCCGGCGGTCTGCACCATCGTCAACCACTACATCGAGACAAGCACGGTCAACTTCCGTACCGAGCCGCAGGAACCGCAGGAGTGGACGGACGACCTCGTCCGTCTGCGGGAGCGCTATCCCTGGCTCGTCGCCGAGGTGGACGGCGAGGTCGCCGGCATCGCCTACGCGGGCCCCTGGAAGGCACGCAACGCCTACGACTGGACGGCCGAGTCGACCGTGTACGTCTCCCCCCGCCACCAGCGGACGGGACTGGGCTCCACGCTCTACACCCACCTGCTGAAGTCCCTGGAGGCACAGGGCTTCAAGAGCGTGGTCGCTGTCATCGGGCTGCCCAACGACCCGAGCGTGCGCATGCACGAGGCGCTCGGATATGCCCCCCGCGGCATGCTGCGGGCGGCCGGCTTCAAGCACGGGAACTGGCATGACGTGGGTTTCTGGCAGCTGGACTTCAGCCTGCCGGTACCGCCCCGTCCGGTCCTGCCCGTCACCGAGATCTGAGATCACGCGTTCTAGTCCGCAAAAATCACCAGTCTCTCTCTACAAATCTATCTCTCTCTATTTTTCTCCAGAATAATGTGTGAGTAGTTCCCAGATAAGGGAATTAGGGTTCTTATAGGGTTTCGCTCATGTGTTGAGCATATAAGAAACCCTTAGTATGTATTTGTATTTGTAAAATACTTCTATCAATAAAATTTCTAATTCCTAAAACCAAAATCCAGTGACCTGCAGGCATGCAAGCTGATCCACTAGAGGCCATGGCGGCCGCGTCGAGCGATCTAGTAACATAGATGACACCGCGCGCGATAATTTATCCTAGTTTGCGCGCTATATTTTGTTTTCTATCGCGTATTAAATGTATAATTGCGGGACTCTAATCATAAAAACCCATCTCATAAATAACGTCATGCATTACATGTTAATTATTACATGCTTAACGTAATTCAACAGAAATTATATGATAATCATCGCAAGACCGGCAACAGGATTCAATCTTAAGAAACTTTATTGCCAAATGTTTGAACGATCGGGGAAATTCGAGTCGACACGCGTAAGCTTCAGCTGCTGCAGGCTCGAGGAGCTCGTCTAGAGGATCGCTCGAGTTATCAGTCGGCCGCGAAGTTGATCTCGCCGTTGTTGAACTTCCTCCTCACCTCCTCCAGGGTCAGGGTGCCGGCCTTGATCATCTCGCCGCCGATCAGGAGCTCCTCCACGGACAGCACGGCGCCGTTGCAGTTGGTGATGTGGTTCAGCCTGGTCAGCTGGGCCTTGTAGTTGCCCTTGAAGTGGCCGGACACGAACAGGAACTTGAACTCGGTCACGCTGGAGGGGTACACCTTCCACCACTCGTTGGGGTTGATGTGCTTGTTCCTGGTCTGGTTCTCCTCCACGTACCTCTGCATTTCGTCGGCCTGGCCGATGGGCAGGTTGTAGCCGCCGGAGTAGGCCTTGGTGTCCACGATCACGCCGTAGTCGATGGGGGAGCCCACGGTGTAGATGGCGCCGTCGGGCTTCCTGGAGCCGCCCAGGTGCTTGCCCCTGTAGCCGTACACCTTCATGAAGAACTCCATCACCTTCATCTCCAGGATACGGTCCTGGGTGCTGTTCCGGGCGATCTCGATCAGCTCGATGTACTCGTGGGGCACGTACTTCAGCTTGTGCCTCAACTCGGATTTCTTCTCCTCCAGCTCGGACTTCACCAGCTGGGAACGGCTGATAGGATCCCCGCCACTTCCGCTGCCACTCGGATCAGGGCGAGATAACTGGGCAACAATGCTCTCCAGCGCCGGCCTGCCGCCGCCATTGCTGGCGATGGCCACCACCTGCTGAGGGGTCAAGCCGTGGGCCTGGCACAGCACCGGCAACAGCCGCTGGACCGTCTCCAGCGCCTGCTTGCCACCGCCATTGCTGGCGATGGCCACCACCTGCTGGGGGGTCAAGCCGTGGGCCTGGCACAGCACCGGCAACAGCCGCTGGACCGTCTCCAGCGCCTGCTTGCCGCCATCGTGGCTGGCGATGGCCACCACCTGCTCCGGGGTCAAGCCGTGGGCCTGGCACAGCACCGGCAACAGCCGCTGGACCGTCTCCAGCGCCTGCTTGCCACCATTATTGCTGGCGATGGCCACCACCTGCTGGGGGGTCAAGCCGTGGGCCTGGCACAGCACCGGCAACAGCGCCTGCACCGTCTCCAGCGCCTGCTTGCCACCAATATTGCTGGCGATGGCCACCACCTGCTCCGGGGTCAAGCCGTGGGCCTGGCACAGCACCGGCAACAGCCGCTGGACCGTCTCCAGCGCCTGCTTGCCACCATTATTGCTGGCGATGGCCACCACCTGCTGGGGGGTCAAGCCGTGGGCCTGGCACAGCACCGGCAACAGCCGCTGGACCGTCTCCAGCGCCTGCTTGCCGCCATCGTGGCTGGCGATGGCCACCACCTGCTCCGGGGTCAAGCCGTGGGCCTGGCACAGCACCGGCAACAGCCGCTGGACCGTCTCCAGCGCCTGCTTGCCACCGCCATTGCTGGCGATGGCCACCACCTGCTGGGGGGTCAAGCCGTGGGCCTGGCACAGCACCGGCAACAGCGCCTGCACCGTCTCCAGCGCCTGCTTGCCACCAATATTGCTGGCGATGGCCACCACCTGCTCCGGGGTCAAGCCGTGGGCCTGGCACAGCACCGGCAACAGCCGCTGGACCGTCTCCAGCGCCTGCTTGCCGCCATCGTGGCTGGCGATGGCCACCACCTGCTCCGGGGTCAAGCCGTGGGCCTGGCACAGCACCGGCAACAGCCGCTGGACCGTCTCCAGCGCCTGCTTGCCACCATTATTGCTGGCGATGGCCACCACCTGCTGGGGGGTCAAGCCGTGGGCCTGGCACAGCACCGGCAACAGCCGCTGGACCGTCTCCAGCGCCTGCTTGCCGCCATCGTGGCTGGCGATGGCCACCACCTGCTCCGGGGTCAAGCCGTGGGCCTGGCACAGCACCGGCAACAGCCGCTGGACCGTCTCCAGCGCCTGCTTGCCGCCATCGTGGCTGGCGATGGCCACCACCTGCTCCGGGGTCAAGCCGTGGGCCTGGCACAGCACCGGCAACAGCGCCTGCACCGTCTCCAGCGCCTGCTTGCCACCAATATTGCTGGCGATGGCCACCACCTGCTCCGGGGTCAAGCCGTGGGCCTGGCACAGCACCGGCAACAGCGCCTGCACCGTCTCCAGCGCCTGCTTGCCACCAATATTGCTGGCGATGGCCACCACCTGCTCCGGGGTCAAGCCGTGGGCCTGGCACAGCACCGGCAACAGCCGCTGGACCGTCTCCAGCGCCTGCTTGCCACCATTATTGCTGGCGATGGCCACCACCTGCTGGGGGGTCAAGTTGAGCGGGGCACCCGTCAGTGCATTGCGCCATGCATGCACTGCCTCCACTGCGGTCACGCCGCCACGTTTTGCAATCTTGAGAAGTTGGCCTGTGTCCAACTGTAACGGTGGACCTCTCAACTCTCCCGCCACCGTGAGCAAGGCCTCCAGAGCGCGTGCGCCGGACCACTGTTTGCCGACGCCAACGATCGCTTCGTGTGTCGCCTCTGGCAACGCTGCGATCATGTCCTGATACTTGACAGCGACGGTCCCTAACGCTGCCGGGTGTTGGCTTAACGCAACGATGTGCGCGTGTGTAAACCCGTGGCCGACCAGTGCCTCGTGGTGCTGCGCCACTGTCGAACGAACCTTCGGTTTGATCTTCTCCTGTTGCTGCTGGCTGTAGCCGAGCGTGCGTAGATCCACCTGCGCCGCCGGCGAAGCGTCGGAGGGTTGCGCAGCACGTCGTCGCGGCGCCGGCTTGGCGCGCGGGGGCCGCGCGGCAGTGACAGCCACGCGCATGGTGGGTGGGGGGGCGTCGGCTGCCCGCAGACCCGATTGCACCTCATCCCACTCGCCTGTGGCAGCCTCTGTATGGTGAGCGCCGAAGGGAGGCAATGAATCAAAAAGCGATGTATTAAAAAGTGACGGATCGAACTGACGTAACAGGTCACTGAAGCTGCCCGCCGAGAACGCAGGTGAGGGGGCAGGGGGAGATGGCAGCCGGGTCCGGGACATCGTCCGCCGAGCCGGCAAGCCATCCAGGGGGCCGCCGGCAGGCGGAGACACCCCACGATCTGCAGTCGGCTGAACCCCATCGGGTTGGGGTCCGGGCAGAAGCTCGCGGGCAGGACTTGGTGTGCGCGAACGAATGGGGTCGGCCATGGTGGCGGCTTGGCGCGTGACAGCGCTGACCTTTCTCTTCTTCTTTGGAGCCATGGCGCGCCGAACTAGTACCTGCAGAAGTAACACCAAACAACAGGGTGAGCATCGACAAAAGAAACAGTACCAAGCAAATAAATAGCGTATGAAGGCAGGGCTAAAAAAATCCACATATAGCTGCTGCATATGCCATCATCCAAGTATATCAAGATCAAAATAATTATAAAACATACTTGTTTATTATAATAGATAGGTACTCAAGGTTAGAGCATATGAATAGATGCTGCATATGCCATCATGTATATGCATCAGTAAAACCCACATCAACATGTATACCTATCCTAGATCGATCCCGTCTGCGGAACGGCTAGAGCCATCCCAGGATTCCCCAAAGAGAAACACTGGCAAGTTAGCAATCAGAACGTGTCTGACGTACAGGTCGCATCCGTGTACGAACGCTAGCAGCACGGATCTAACACAAACACGGATCTAACACAAACATGAACAGAAGTAGAACTACCGGGCCCTAACCATGGACCGGAACGCCGATCTAGAGAAGGTAGAGAGGGGGGGGGGGGGAGGACGAGCGGCGTACCTTGAAGCGGAGGTGCCGACGGGTGGATTTGGGGGAGATCTGGTTGTGTGTGTGTGCGCTCCGAACAACACGAGGTTGGGGAAAGAGGGTGTGGAGGGGGTGTCTATTTATTACGGCGGGCGAGGAAGGGAAAGCGAAGGAGCGGTGGGAAAGGAATCCCCCGTAGCTGCCGGTGCCGTGAGAGGAGGAGGAGGCCGCCTGCCGTGCCGGCTCACGTCTGCCGCTCCGCCACGCAATTTCTGGATGCCGACAGCGGAGCAAGTCCAACGGTGGAGCGGAACTCTCGAGAGGGGTCCAGAGGCAGCGACAGAGATGCCGTGCCGTCTGCTTCGCTTGGCCCGACGCGACGCTGCTGGTTCGCTGGTTGGTGTCCGTTAGACTCGTCGATCGACGGCGTTTAACAGGCTGGCATTATCTACTCGAAACAAGAAAAATGTTTCCTTAGTTTTTTTAATTTCTTAAAGGGTATTTGTTTAATTTTTAGTCACTTTATTTTATTCTATTTTATATCTAAATTATTAAATAAAAAAACTAAAATAGAGTTTTAGTTTTCTTAATTTAGAGGCTAAAATAGAATAAAATAGATGTACTAAAAAAATTAGTCTATAAAAACCATTAACCCTAAACCCTAAATGGATGTACTAATAAAATGGATGAAGTATTATATAGGTGAAGCTATTTGCAAAAAAAAAGGAGAACACATGCACACTAAAAAGATAAAACTGTAGAGTCCTGTTGTCAAAATACTCAATTGTCCTTTAGACCATGTCTAACTGTTCATTTATATGATTCTCTAAAACACTGATATTATTGTAGTACTATAGATTATATTATTCGTAGAGTAAAGTTTAAATATATGTATAAAGATAGATAAACTGCACTTCAAACAAGTGTGACAAAAAAAATATGTGGTAATTTTTTATAACTTAGACATGCAATGCTCATTATCTCTAGAGAGGGGCACGACCGGGTCACGCTGCACTGCAGCCTAGTAAGGCCTTAAGGGCCAGATCTTGGGCCCGGTACCCGATCAGATTGTCGTTTCCCGCCTTCGGTTTAAACTATCAGTGTTTGACAGGATATATTGGCGGGTAAACCTAAGAGAAAAGAGCGTTTATTAGAATAATCGGATATTTAAAAGGGCGTGAAAAGGTTTATCCGTTCGTCCATTTGTATGTGCATGCCAACCACAGGGTTCCCCTCGGGAGTGCTTGGCATTCCGTGCGATAATGACTTCTGTTCAACCACCCAAACGTCGGAAAGCCTGACGACGGAGCAGCATTCCAAAAAGATCCCTTGGCTCGTCTGGGTCGGCTAGAAGGTCGAGTGGGCTGCTGTGGCTTGATCCCTCAACGCGGTCGCGGACGTAGCGCAGCGCCGAAAAATCCTCGATCGCAAATCCGACGCTGTCGAAAAGCGTGATCTGCTTGTCGCTCTTTCGGCCGACGTCCTGGCCAGTCATCACGCGCCAAAGTTCCGTCACAGGATGATCTGGCGCGAGTTGCTGGATCTCGCCTTCAATCCGGGTCTGTGGCGGGAACTCCACGAAAATATCCGAACGCAGCAAGATATCGCGGTGCATCTCGGTCTTGCCTGGGCAGTCGCCGCCGACGCCGTTGATGTGGACGCCGAAAAGGATCTAGGTGAAGATCCTTTTTGATAATCTCATGACCAAAATCCCTTAACGTGAGTTTTCGTTCCACTGAGCGTCAGACCCCGTAGAAAAGATCAAAGGATCTTCTTGAGATCCTTTTTTTCTGCGCGTAATCTGCTGCTTGCAAACAAAAAAACCACCGCTACCAGCGGTGGTTTGTTTGCCGGATCAAGAGCTACCAACTCTTTTTCCGAAGGTAACTGGCTTCAGCAGAGCGCAGATACCAAATACTGTTCTTCTAGTGTAGCCGTAGTTAGGCCACCACTTCAAGAACTCTGTAGCACCGCCTACATACCTCGCTCTGCTAATCCTGTTACCAGTGGCTGCTGCCAGTGGCGATAAGTCGTGTCTTACCGGGTTGGACTCAAGACGATAGTTACCGGATAAGGCGCAGCGGTCGGGCTGAACGGGGGGTTCGTGCACACAGCCCAGCTTGGAGCGAACGACCTACACCGAACTGAGATACCTACAGCGTGAGCTATGAGAAAGCGCCACGCTTCCCGAAGGGAGAAAGGCGGACAGGTATCCGGTAAGCGGCAGGGTCGGAACAGGAGAGCGCACGAGGGAGCTTCCAGGGGGAAACGCCTGGTATCTTTATAGTCCTGTCGGGTTTCGCCACCTCTGACTTGAGCGTCGATTTTTGTGATGCTCGTCAGGGGGGCGGAGCCTATGGAAAAACGCCAGCAACGCGGCCTTTTTACGGTTCCTGGCCTTTTGCTGGCCTTTTGCTCACATGTTCTTTCCTGCGTTATCCCCTGATTCTGTGGATAACCGATTACCGCCTTTGAGTGAGCTGATACCGCTCGCCGCAGCCGAACGACCGAGCGCAGCGAGTCAGTGAGCGAGGAAGCGGAAGAGCGCCTGATGCGGTATTTTCTCCTTACGCATCTGTGCGGTATTTCACACCGCATATGGTGCACTCTCAGTACAATCTGCTCTGATGCCGCATAGTTAAGCCAGTATACACTCCGCTATCGCTACGTGACTGGGTCATGGCTGCGCCCCGACACCCGCCAACACCCGCTGACGCGCCCTGACGGGCTTGTCTGCTCCCGGCATCCGCTTACAGACAAGCTGTGACCGTCTCCGGGAGCTGCATGTGTCAGAGGTTTTCACCGTCATCACCGAAACGCGCGAGGCAGGGGTACGTCGAGGTCGATCCAACCCCTCCGCTGCTATAGTGCAGTCGGCTTCTGACGTTCAGTGCAGCCGTCTTCTGAAAACGACATGTCGCACAAGTCCTAAGTTACGCGACAGGCTGCCGCCCTGCCCTTTTCCTGGCGTTTTCTTGTCGCGTGTTTTAGTCGCATAAAGTAGAATACTTGCGACTAGAACCGGAGACATTACGCCATGAACAAGAGCGCCGCCGCTGGCCTGCTGGGCTATGCCCGCGTCAGCACCGACGACCAGGACTTGACCAACCAACGGGCCGAACTGCACGCGGCCGGCTGCACCAAGCTGTTTTCCGAGAAGATCACCGGCACCAGGCGCGACCGCCCGGAGCTGGCCAGGATGCTTGACCACCTACGCCCTGGCGACGTTGTGACAGTGACCAGGCTAGACCGCCTGGCCCGCAGCACCCGCGACCTACTGGACATTGCCGAGCGCATCCAGGAGGCCGGCGCGGGCCTGCGTAGCCTGGCAGAGCCGTGGGCCGACACCACCACGCCGGCCGGCCGCATGGTGTTGACCGTGTTCGCCGGCATTGCCGAGTTCGAGCGTTCCCTAATCATCGACCGCACCCGGAGCGGGCGCGAGGCCGCCAAGGCGCGAGGCGTGAAGTTTGGCCCCCGCCCTACCCTCACCCCGGCACAGATCGCGCACGCCCGCGAGCTGATCGACCAGGAAGGCCGCACCGTGAAAGAGGCGGCTGCACTGCTTGGCGTGCATCGCTCGACCCTGTACCGCGCACTTGAGCGCAGCGAGGAAGTGACGCCCACCGAGGCCAGGCGGCGCGGTGCCTTCCGTGAGGACGCATTGACCGAGGCCGACGCCCTGGCGGCCGCCGAGAATGAACGCCAAGAGGAACAAGCATGAAACCGCACCAGGACGGCCAGGACGAACCGTTTTTCATTACCGAAGAGATCGAGGCGGAGATGATCGCGGCCGGGTACGTGTTCGAGCCGCCCGCGCACGTCTCAACCGTGCGGCTGCATGAAATCCTGGCCGGTTTGTCTGATGCCAAGCTCGCGGCCTGGCCGGCGAGCTTGGCCGCTGAAGAAACCGAGCGCCGCCGTCTAAAAAGGTGATGTGTATTTGAGTAAAACAGCTTGCGTCATGCGGTCGCTGCGTATATGATGCGATGAGTAAATAAACAAATACGCAAGGGGAACGCATGAAGGTTATCGCTGTACTTAACCAGAAAGGCGGGTCAGGCAAGACGACCATCGCAACCCATCTAGCCCGCGCCCTGCAACTCGCCGGGGCCGATGTTCTGTTAGTCGATTCCGATCCCCAGGGCAGTGCCCGCGATTGGGCGGCCGTGCGGGAAGATCAACCGCTAACCGTTGTCGGCATCGACCGCCCGACGATTGACCGCGACGTGAAGGCCATCGGCCGGCGCGACTTCGTAGTGATCGACGGAGCGCCCCAGGCGGCGGACTTGGCTGTGTCCGCGATCAAGGCAGCCGACTTCGTGCTGATTCCGGTGCAGCCAAGCCCTTACGACATATGGGCCACCGCCGACCTGGTGGAGCTGGTTAAGCAGCGCATTGAGGTCACGGATGGAAGGCTACAAGCGGCCTTTGTCGTGTCGCGGGCGATCAAAGGCACGCGCATCGGCGGTGAGGTTGCCGAGGCGCTGGCCGGGTACGAGCTGCCCATTCTTGAGTCCCGTATCACGCAGCGCGTGAGCTACCCAGGCACTGCCGCCGCCGGCACAACCGTTCTTGAATCAGAACCCGAGGGCGACGCTGCCCGCGAGGTCCAGGCGCTGGCCGCTGAAATTAAATCAAAACTCATTTGAGTTAATGAGGTAAAGAGAAAATGAGCAAAAGCACAAACACGCTAAGTGCCGGCCGTCCGAGCGCACGCAGCAGCAAGGCTGCAACGTTGGCCAGCCTGGCAGACACGCCAGCCATGAAGCGGGTCAACTTTCAGTTGCCGGCGGAGGATCACACCAAGCTGAAGATGTACGCGGTACGCCAAGGCAAGACCATTACCGAGCTGCTATCTGAATACATCGCGCAGCTACCAGAGTAAATGAGCAAATGAATAAATGAGTAGATGAATTTTAGCGGCTAAAGGAGGCGGCATGGAAAATCAAGAACAACCAGGCACCGACGCCGTGGAATGCCCCATGTGTGGAGGAACGGGCGGTTGGCCAGGCGTAAGCGGCTGGGTTGTCTGCCGGCCCTGCAATGGCACTGGAACCCCCAAGCCCGAGGAATCGGCGTGAGCGGTCGCAAACCATCCGGCCCGGTACAAATCGGCGCGGCGCTGGGTGATGACCTGGTGGAGAAGTTGAAGGCGGCGCAGGCCGCCCAGCGGCAACGCATCGAGGCAGAAGCACGCCCCGGTGAATCGTGGCAAGCGGCCGCTGATCGAATCCGCAAAGAATCCCGGCAACCGCCGGCAGCCGGTGCGCCGTCGATTAGGAAGCCGCCCAAGGGCGACGAGCAACCAGATTTTTTCGTTCCGATGCTCTATGACGTGGGCACCCGCGATAGTCGCAGCATCATGGACGTGGCCGTTTTCCGTCTGTCGAAGCGTGACCGACGAGCTGGCGAGGTGATCCGCTACGAGCTTCCAGACGGGCACGTAGAGGTTTCCGCAGGGCCGGCCGGCATGGCGAGTGTGTGGGATTACGACCTGGTACTGATGGCGGTTTCCCATCTAACCGAATCCATGAACCGATACCGGGAAGGGAAGGGAGACAAGCCCGGCCGCGTGTTCCGTCCACACGTTGCGGACGTACTCAAGTTCTGCCGGCGAGCCGATGGCGGAAAGCAGAAAGACGACCTGGTAGAAACCTGCATTCGGTTAAACACCACGCACGTTGCCATGCAGCGTACGAAGAAGGCCAAGAACGGCCGCCTGGTGACGGTATCCGAGGGTGAAGCCTTGATTAGCCGCTACAAGATCGTAAAGAGCGAAACCGGGCGGCCGGAGTACATCGAGATCGAGCTAGCTGATTGGATGTACCGCGAGATCACAGAAGGCAAGAACCCGGACGTGCTGACGGTTCACCCCGATTACTTTTTGATCGATCCCGGCATCGGCCGTTTTCTCTACCGCCTGGCACGCCGCGCCGCAGGCAAGGCAGAAGCCAGATGGTTGTTCAAGACGATCTACGAACGCAGTGGCAGCGCCGGAGAGTTCAAGAAGTTCTGTTTCACCGTGCGCAAGCTGATCGGGTCAAATGACCTGCCGGAGTACGATTTGAAGGAGGAGGCGGGGCAGGCTGGCCCGATCCTAGTCATGCGCTACCGCAACCTGATCGAGGGCGAAGCATCCGCCGGTTCCTAATGTACGGAGCAGATGCTAGGGCAAATTGCCCTAGCAGGGGAAAAAGGTCGAAAAGGTCTCTTTCCTGTGGATAGCACGTACATTGGGAACCCAAAGCCGTACATTGGGAACCGGAACCCGTACATTGGGAACCCAAAGCCGTACATTGGGAACCGGTCACACATGTAAGTGACTGATATAAAAGAGAAAAAAGGCGATTTTTCCGCCTAAAACTCTTTAAAACTTATTAAAACTCTTAAAACCCGCCTGGCCTGTGCATAACTGTCTGGCCAGCGCACAGCCGAAGAGCTGCAAAAAGCGCCTACCCTTCGGTCGCTGCGCTCCCTACGCCCCGCCGCTTCGCGTCGGCCTATCGCGGCCGCTGGCCGCTCAAAAATGGCTGGCCTACGGCCAGGCAATCTACCAGGGCGCGGACAAGCCGCGCCGTCGCCACTCGACCGCCGGCGCCCACATCAAGGCACCGGTGGGTATGCCTGACGATGCGTGGAGACCGAAACCTTGCGCTCGTTCGCCAGCCAGGACAGAAATGCCTCGACTTCGCTGCTGCCCAAGGTTGCCGGGTGACGCACACCGTGGAAACGGATGAAGGCACGAACCCAGTGGACATAAGCCTGTTCGGTTCGTAAGCTGTAATGCAAGTAGCGTATGCGCTCACGCAACTGGTCCAGAACCTTGACCGAACGCAGCGGTGGTAACGGCGCAGTGGCGGTTTTCATGGCTTGTTATGACTGTTTTTTTGGGGTACAGTCTATGCCTCGGGCATCCAAGCAGCAAGCGCGTTACGCCGTGGGTCGATGTTTGATGTTATGGAGCAGCAACGATGTTACGCAGCAGGGCAGTCGCCCTAAAACAAAGTTAAACATCATGAGGGAAGCGGTGATCGCCGAAGTATCGACTCAACTATCAGAGGTAGTTGGCGTCATCGAGCGCCATCTCGAACCGACGTTGCTGGCCGTACATTTGTACGGCTCCGCAGTGGATGGCGGCCTGAAGCCACACAGTGATATTGATTTGCTGGTTACGGTGACCGTAAGGCTTGATGAAACAACGCGGCGAGCTTTGATCAACGACCTTTTGGAAACTTCGGCTTCCCCTGGAGAGAGCGAGATTCTCCGCGCTGTAGAAGTCACCATTGTTGTGCACGACGACATCATTCCGTGGCGTTATCCAGCTAAGCGCGAACTGCAATTTGGAGAATGGCAGCGCAATGACATTCTTGCAGGTATCTTCGAGCCAGCCACGATCGACATTGATCTGGCTATCTTGCTGACAAAAGCAAGAGAACATAGCGTTGCCTTGGTAGGTCCAGCGGCGGAGGAACTCTTTGATCCGGTTCCTGAACAGGATCTATTTGAGGCGCTAAATGAAACCTTAACGCTATGGAACTCGCCGCCCGACTGGGCTGGCGATGAGCGAAATGTAGTGCTTACGTTGTCCCGCATTTGGTACAGCGCAGTAACCGGCAAAATCGCGCCGAAGGATGTCGCTGCCGACTGGGCAATGGAGCGCCTGCCGGCCCAGTATCAGCCCGTCATACTTGAAGCTAGACAGGCTTATCTTGGACAAGAAGAAGATCGCTTGGCCTCGCGCGCAGATCAGTTGGAAGAATTTGTCCACTACGTGAAAGGCGAGATCACCAAGGTAGTCGGCAAATAATGTCTAACAATTCGTTCAAGCCGACGCCGCTTCGCGGCGCGGCTTAACTCAAGCGTTAGATGCACTAAGCACATAATTGCTCACAGCCAAACTATCAGGTCAAGTCTGCTTTTATTATTTTTAAGCGTGCATAATAAGCCCTACACAAATTGGGAGATATATCATGAAAGGCTGGCTTTTTCTTGTTATCGCAATAGTTGGCGAAGTAATCGCAACATAGCTTGCTTGGTCGTTCCGCGTGAACGTCGGCTCGATTGTACCTGCGTTCAAATACTTTGCGATCGTGTTGCGCGCCTGCCCGGTGCGTCGGCTGATCTCACGGATCGACTGCTTCTCTCGCAACGCCATCCGACGGATGATGTTTAAAAGTCCCATGTGGATCACTCCGTTGCCCCGTCGCTCACCGTGTTGGGGGGAAGGTGCACATGGCTCAGTTCTCAATGGAAATTATCTGCCTAACCGGCTCAGTTCTGCGTAGAAACCAACATGCAAGCTCCACCGGGTGCAAAGCGGCAGCGGCGGCAGGATATATTCAATTGTAAATGGCTTCATGTCCGGGAAATCTACATGGATCAGCAATGAGTATGATGGTCAATATGGAGAAAAAGAAAGAGTAATTACCAATTTTTTTTCAATTCAAAAATGTAGATGTCCGCAGCGTTATTATAAAATGAAAGTACATTTTGATAAAACGACAAATTACGATCCGTCGTATTTATAGGCGAAAGCAATAAACAAATTATTCTAATTCGGAAATCTTTATTTCGACGTGTCTACATTCACGTCCAAATGGGGGCTTAGATGAGAAACTTCACGATCGGC
